# Endothelial STING-JAK1 interaction promotes tumor vasculature normalization and antitumor immunity

**DOI:** 10.1172/JCI180622

**Published:** 2025-01-16

**Authors:** Huanling Zhang, Zining Wang, Jiaxin Wu, Yong-Qiang Zheng, Qi Zhao, Shuai He, Hang Jiang, Chang Jiang, Tiantian Wang, Yongxiang Liu, Lei Cui, Hui Guo, Jiahong Yi, Huan Jin, Chunyuan Xie, Mengyun Li, Jiahui Li, Xiaojuan Wang, Liangping Xia, Xiao-Shi Zhang, Xiaojun Xia

**Affiliations:** 1 State Key Laboratory of Oncology in South China, Guangdong Provincial Clinical Research Center for Cancer, Sun Yat-sen University Cancer Center, Guangzhou, China.; 2 Guangzhou Institute of Clinical Medicine, Guangzhou First People’s Hospital, Guangzhou, China.; 3 Department of Biotherapy, Sun Yat-sen University Cancer Center, Guangzhou, China.; 4 Department of Oncology, Affiliated Hospital of Guizhou Medical University, Guiyang, Guizhou, China.; 5 VIP region, Sun Yat-sen University Cancer Center, Guangzhou, China.; 6 MOE Key Laboratory of Gene Function and Regulation, Guangdong Province Key Laboratory of Pharmaceutical Functional Genes, State Key Laboratory of Biocontrol, School of Life Sciences, Sun Yat-sen University, Guangzhou, China.; 7 School of Food Science and Technology, Dalian Polytechnic University, Dalian, China.; 8 Hainan Academy of Medical Sciences, Hainan Medical University, Haikou, China.

**Keywords:** Immunology, Oncology, Cancer immunotherapy

## Abstract

Stimulator of interferon genes (STING) agonists have been developed and tested in clinical trials for their antitumor activity. However, the specific cell population(s) responsible for such STING activation–induced antitumor immunity have not been completely understood. In this study, we demonstrated that endothelial STING expression was critical for STING agonist–induced antitumor activity. STING activation in endothelium promoted vessel normalization and CD8^+^ T cell infiltration — which required type I IFN (IFN-I) signaling— but not IFN-γ or CD4^+^ T cells. Rather than an upstream adaptor for inducing IFN-I signaling, STING acted downstream of interferon-α/β receptor (IFNAR) in endothelium for the JAK1-STAT signaling activation. Mechanistically, IFN-I stimulation induced JAK1-STING interaction and promoted JAK1 phosphorylation, which involved STING palmitoylation at the Cysteine 91 site but not its C-terminal tail (CTT) domain. Endothelial STING and JAK1 expression was significantly associated with immune cell infiltration in patients with cancer, and STING palmitoylation level correlated positively with CD8^+^ T cell infiltration around STING-positive blood vessels in tumor tissues from patients with melanoma. In summary, our findings uncover a previously unrecognized function of STING in regulating JAK1/STAT activation downstream of IFN-I stimulation and provide a new insight for future design and clinical application of STING agonists for cancer therapy.

## Abstract 2

The current knowledge about STING-IFN-I signaling considers STING as an upstream adaptor for IFN-I induction. However, our study identifies a previously unrecognized function of STING in regulating JAK1/STAT activation downstream of IFN-I stimulation in endothelial cells.

## Graphical abstract

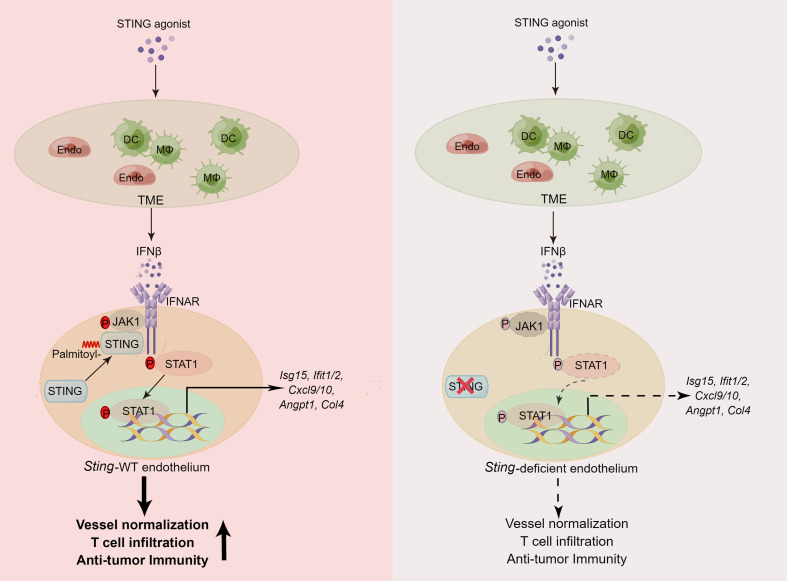

## Introduction

The stimulator of interferon genes (STING) protein resides in the ER (1). Upon binding with its natural ligand, cyclic GMP-AMP (cGAMP), which is synthesized by cGAMP synthase (cGAS), STING becomes activated and translocates to the Golgi to recruit and activate TANK-binds kinase 1 (TBK1) and IFN regulatory factor 3 (IRF3), inducing type I IFN (IFN-I) signaling, nuclear factor-κB (NF-κB) activation and inflammation responses (2, 3). The full STING protein structure contains a transmembrane (TM) domain, a CDN binding domain (CBD), and a C-terminal tail domain (CTT) essential for recruiting TBK1 and IRF3 for phosphorylation and activation (4). After activation, STING transfers to the Golgi and undergoes palmitoylation at cysteine 88/91 to facilitate STING clustering formation and signal transduction (5). STING signaling plays a critical role in connecting innate and adaptive immunity, as the robust production of IFN-I enhances CD8^+^ T cell cross priming by tumor antigens (6, 7). On the other hand, STING has been identified as a driver of chronic inflammation and functional decline during ageing or as a cell-intrinsic metabolic checkpoint restricting aerobic glycolysis by targeting Hexokinase 2 (8, 9).

Recently, various STING agonists have been developed for cancer therapy by intratumoral injection or systemic administration (10–13). The STING agonists, such as MIW815 (ADU-S100) and MK-1454, have demonstrated strong antitumor efficacy in preclinical studies but failed to elicit antitumor immune responses or immune infiltration in patients with advanced solid tumors or lymphomas (14, 15). A series of studies reported the synergistic effect of STING agonists with other immunotherapy treatments for inducing strong systemic antitumor immune response (16–18), whereas only minimal antitumor responses were detected in clinical trials (19). A major obstacle for inducing strong antitumor immunity by STING agonists is the tumor microenvironment, which is a complex ecosystem. The tumor microenvironment contains not only tumor cells but also tumor vasculatures, immune cells, fibroblasts, extracellular matrix, and more. (20, 21). STING agonists appear to be capable of activating effective IFN-I signaling in many cell types within the tumor microenvironment, such as endothelial cells, macrophages, DCs, and T cells (22–24). However, the specific cell type(s) responsible for the STING agonist–induced antitumor activity in vivo has remained incompletely understood.

Previous studies have shown that the antitumor immunity induced by intratumoral STING agonists was dependent on host IFN-I signaling and CD8^+^ T cells (7, 25). Nevertheless, the cellular source of IFN-I in the tumor environment remained controversial. Early studies showed that tumor DNA induced STING activation and IFN-I signaling in DCs, which act as the major cellular source of IFN-β (25, 26). However, another study showed that endothelial cells rather than DCs, were the major source of IFN-β after intratumoral cGAMP injection in a mouse melanoma model (27). Moreover, STING activation in the endothelium was shown to be critical for the normalization of tumor vasculature and T cell infiltration (28, 29), suggesting endothelium as an important target of STING agonists. Furthermore, macrophages seem critical for cGAMP-mediated antitumor immunity, as the antitumor effect was ablated when macrophages were depleted (30), yet another study found a dispensable role of macrophages in STING agonist–induced tumor vasculature normalization and antitumor activity (28). An early study carefully examined the impact of STING signaling magnitude on its antitumor effect and suggested that IFN-I, but not TNF-α, was required for optimal antitumor immune responses mediated by STING agonists (31). However, a recent report showed that tumor-associated myeloid cells secreted TNF-α upon STING activation and induced apoptosis of tumor endothelium to promote tumor immunity (32). Hence, the exact cell type and effector immune molecules responsible for intratumoral STING activation–induced antitumor activity remain unclear.

To define the cell type–specific function of STING in intratumor STING agonist–induced tumor inhibition, we used Cre-flox recombination approach to generate tissue-specific *Sting* (*Tmem173*) gene knockout mouse models. Surprisingly, we found that *Sting* knockout in DCs and macrophages had no effect on antitumor activity, but *Sting* knockout in endothelial cells drastically abolished the antitumor activity of intratumoral STING agonist. Upon IFN-β stimulation, STING in endothelial cells interacted with JAK1 to facilitate JAK1-STAT1 signal transmission and induction of downstream genes for vasculature normalization. The phosphorylation of JAK1 and the palmitoylation of STING at the C91 site, independent of STING CTT domain, was critical for the JAK1-STING interaction. Thus, our findings identify a previously unrecognized role of STING in regulating JAK1-STAT1 signaling downstream of IFN-β and provide a new critical insight for future design and clinical application of STING agonists for cancer therapy.

## Results

### STING agonist–inhibited tumor growth via IFNAR signaling and CD8^+^ T cells.

**Figure 1:**
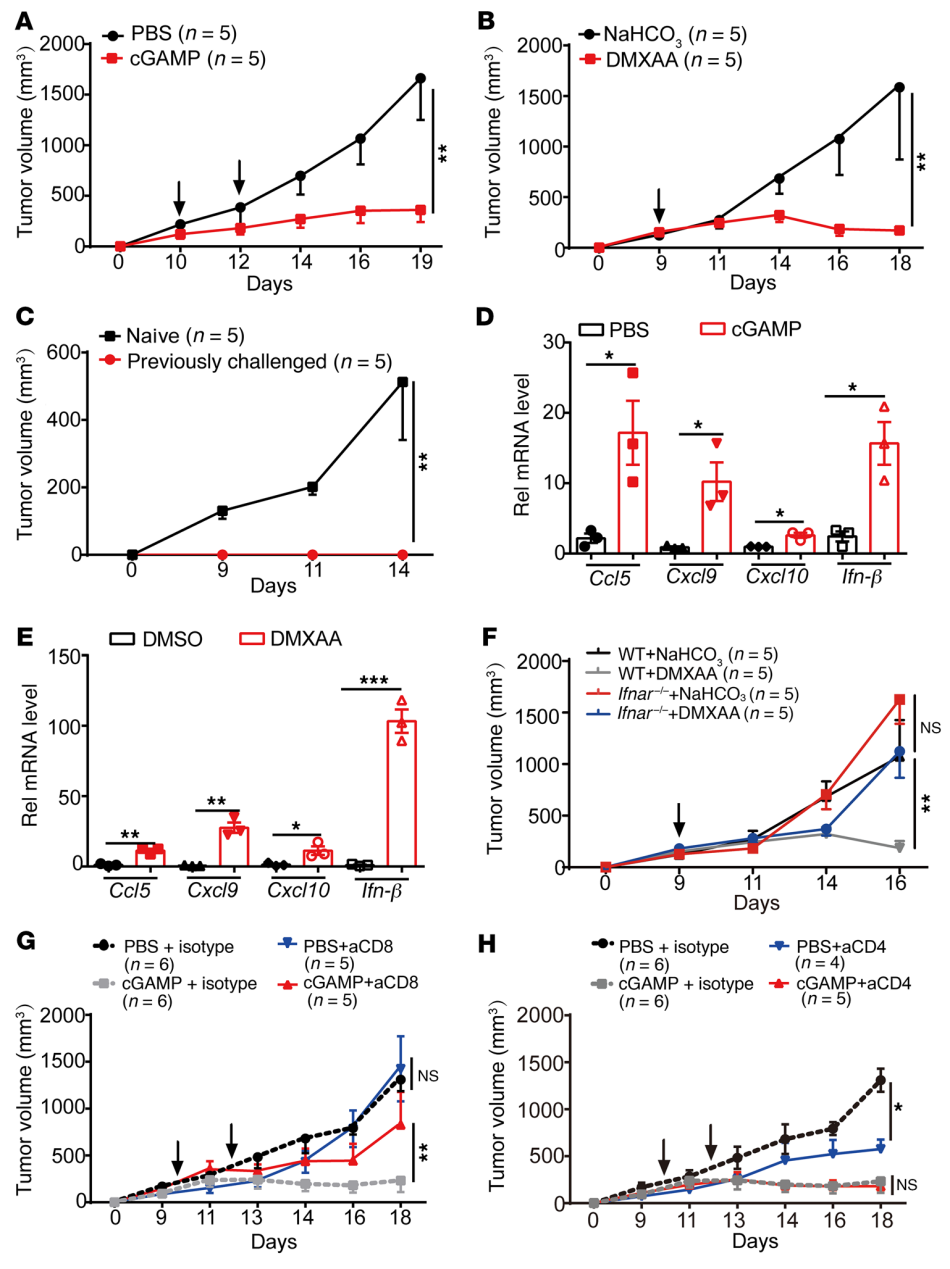
STING agonists inhibit tumor growth depending on IFNAR signaling and CD8^+^ T cells. (**A** and **B**) C57BL/6 mice bearing B16 tumors were treated with intratumoral (i.t.) injection of cGAMP (**A**, 25 μg/mouse) or DMXAA (**B**, 200 μg/mouse) (indicated by the arrow), and tumor sizes were recorded. (**C**) Naive mice and mice that had completely eliminated B16 tumors by DMXAA injection were rechallenged with B16 tumor cells at a distant site, and tumor sizes were measured.(**D** and **E**) Mice bearing B16 tumors were treated with i.t. injection of cGAMP (**D**) or DMXAA (**E**), and B16 tumor tissues were collected 3 hours later for RNA isolation and qRT-PCR analysis. (**F**) WT or *Ifnar^–/–^* mice bearing B16 tumors were treated with i.t. injection of DMXAA (indicated by the arrow), and tumor growth was monitored. (**G** and **H**) C57BL/6 mice bearing B16 tumors were treated with i.t. injection of cGAMP (indicated by the arrow) when tumor size reached 100 mm^3^, and intraperitoneal (i.p.) injection of anti-CD8 mAb (**G**, 100 μg/mouse) or anti-CD4 mAb (**H**, 100 μg/mouse) once every 3 days since day 9. Tumor growth was monitored. The number of mice used in each group is shown in the figure. Data are represented as mean ± SEM. *P < 0.05; **P < 0.01; ***P < 0.001, by 2-way ANOVA with Šidák’s multiple comparisons test (**A**–**C** and **F**–**H**) or by 2-tailed unpaired *t* test (**D** and **E**).

Intratumoral administration of STING agonists has been demonstrated with effective antitumor activity on multiple mouse tumor models (33, 34). We first verified that intratumoral administration of either a strong (DMXAA) or mild STING agonist (cGAMP) both significantly inhibited B16 mouse melanoma growth (Figure 1, A and B). Furthermore, those cured mice rejected the rechallenge of B16 tumor cells at a distant site, indicating an antitumor immune memory response after STING agonist treatment (Figure 1C). In line with previous studies, intratumoral mRNA expression levels of STING downstream genes such as *Ccl5*, *Cxcl9*, *Cxcl10*, and *Ifn-β* were all upregulated in tumors after treatment with cGAMP or DMXAA (Figure 1, D and E). To further examine whether intratumoral STING agonist–induced antitumor effect is dependent on IFN-I or IFN-γ pathway, we then repeated the intratumoral treatment experiments using *Ifnar^–/–^* mice or an IFN-γ blocking antibody pretreatment and found that the antitumor effect was significantly impaired by *Ifnar* deficiency (Figure 1F, and Supplemental Figure 1A; supplemental material available online with this article; https://doi.org/10.1172/JCI180622DS1), though this did not occur after IFN-γ blockade (Supplemental Figure 1B). Finally, to determine whether CD4^+^ or CD8^+^ T cells were required for STING agonist–induced antitumor response, we treated mice with neutralizing antibodies against CD8 and CD4 and found that only CD8^+^ T cell depletion abrogated the antitumor efficacy of STING agonists, while CD4 depletion had no effect (Figure 1, G and H). These results suggest that intratumoral STING agonist administration inhibits tumor growth mainly dependent on IFN-I signaling and CD8^+^ T cells.

### STING expression in endothelial cells is essential for the antitumor effect of STING agonists in MC38 and B16 tumor models.

As STING is expressed on both tumor cells and host cells such as DCs, macrophages, and endothelial cells, we next tested STING activation in which cell type contributed most to the antitumor effect of the intratumoral STING agonist. We generated *Sting* knockout tumor cell lines (B16 and MC38) using CRISPR/Cas9 technology and used these cells for xenograft and treatment experiments. In line with previous findings (35), we also found that *Sting* knockout in tumor cells did not affect xenograft tumor growth nor tumor inhibition by intratumoral DMXAA treatment (Supplemental Figure 2, A and B). These results suggest that STING expression in tumor cells is not required for the antitumor effects of intratumoral STING agonist.

**Figure 2:**
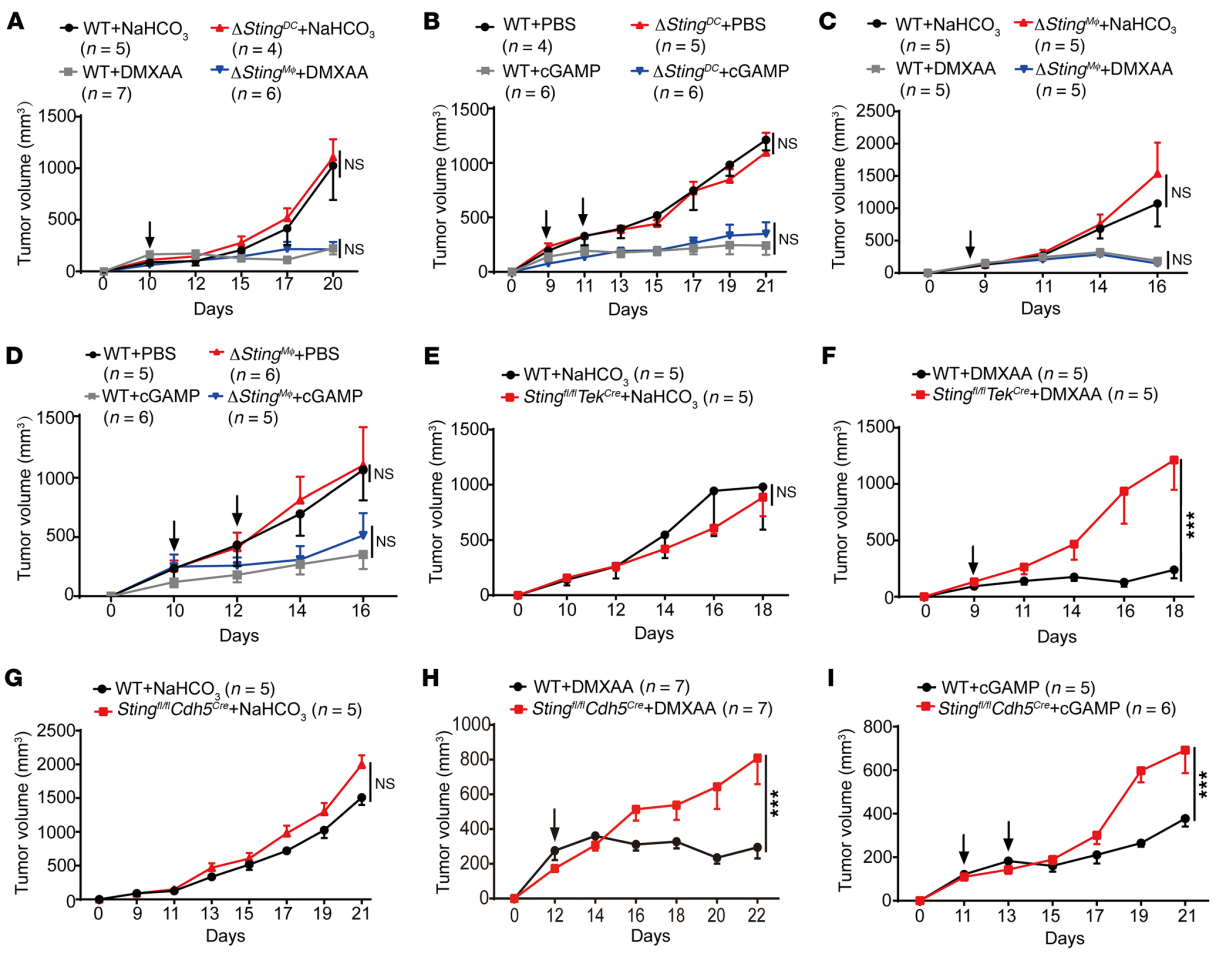
STING expression in endothelial cells is essential for the antitumor effect of STING agonists on MC38 and B16 tumor models. (**A** and **B**) WT or *Sting^fl/fl^/Itgax*-Cre (*ΔSting^DC^*) mice bearing B16 (**A**) or B16-OVA (**B**) tumors were treated with i.t. injection of DMXAA (**A**, 200 μg/mouse) or cGAMP (**B**, 25 μg/mouse), tumor growth was recorded. NaHCO_3_ or PBS vehicle treatment was used as control. (**C** and **D**) WT or *Sting^fl/fl^/LysM*-Cre (*ΔSting^Mϕ^*) mice bearing B16 tumors were treated with i.t. injection of DMXAA (**C**) or cGAMP (**D**) and tumor growth was recorded. (**E** and **F**) WT and *Sting^fl/fl^/Tek*-Cre mice bearing B16 tumors were treated with i.t. injection of vehicle (5% NaHCO_3_). (**E**) or DMXAA (**F**) and tumor growth was recorded. (**G**–**I**) WT or *Sting^fl/fl^/Cdh5*-Cre mice bearing B16 tumors were treated with i.t. injection of vehicle (5% NaHCO_3_) (**G**), DMXAA (**H**), or cGAMP (**I**) and tumor growth was recorded. The timing of STING agonist injections is indicated by arrows. The number of mice used in each group is shown in the Figure. Data are represented as mean ± SEM. ***P < 0.001, by 2-way ANOVA with Šidák’s multiple comparisons test (**A**–**I**).

Next, to examine whether STING in host myeloid cells contributed to the antitumor effects of the STING agonist, we generated DC- and macrophage-specific *Sting* conditional knockout (cKO) mice by crossing *Sting^fl/fl^* mice with *Itgax-Cre* or *LysM-Cre* mice, respectively (Supplemental Figure 2C). The knockout efficiency of STING expression in DCs or macrophages in these mice was confirmed by qPCR and Western blot (Supplemental Figure 2, D and E). As expected, STING agonist–induced downstream chemokine expression was also abrogated in *Sting*^–/–^ DCs or macrophages (Supplemental Figure 2F). Surprisingly, despite the defective chemokine response, the antitumor effect of intratumoral injection of STING agonist was comparable between WT and *Sting*-DC cKO (*Sting^fl/fl^/Itgax-Cre*) mice bearing tumors (B16, B16-OVA, and MC38) (Figure 2, A and B, and Supplemental Figure 2, G and H). Likewise, STING deficiency in macrophages from *Sting*-Mϕ cKO (*Sting^fl/fl^/Lysm-Cre*) mice did not impair the intratumoral STING agonist–induced antitumor effect either (Figure 2, C and D). These results suggest that intratumoral STING activation inhibits tumor growth independent of STING expression in host myeloid cells, including DCs and macrophages.

Previous reports have found that STING activation reprogrammed tumor vasculatures to promote antitumor immunity (27, 28). Thus, we reasoned that STING expression in endothelium might contribute to the antitumor effects of the intratumoral STING activation. To explore this possibility, we first generated endothelium-specific *Sting* KO mice by crossing *Tek-Cre* or tamoxifen-inducible *Cdh5-CreERT2* transgenic mice with *Sting^fl/fl^* mice to obtain *Sting^fl/fl^/Tek-Cre* and *Sting^fl/fl^/Cdh5-Cre* mice (Supplemental Figure 2I), then performed tumor xenograft and treatment experiments using these mice. Tumor growth was comparable between WT and *Sting^fl/fl^/Tek-Cre* mice without treatment (Figure 2E), but the tumor inhibition effect of intratumor STING agonist administration on WT mice was markedly compromised on *Sting^fl/fl^/Tek-Cre* mice (Figure 2F). Similar results were observed on *Sting^fl/fl^/Cdh5-Cre* mice upon intratumor administration of either DMXAA or cGAMP (Figure 2, G–I). These results indicate that, compared to its expression in tumor cells, macrophages, or DCs, STING expression in endothelial cells plays a more prominent role in mediating the antitumor effect of STING agonists.

### Deficiency of STING in endothelial cells impaired the infiltration of CD8^+^ T cells and tumor blood vessel normalization, but not IFN-β production.

**Figure 3:**
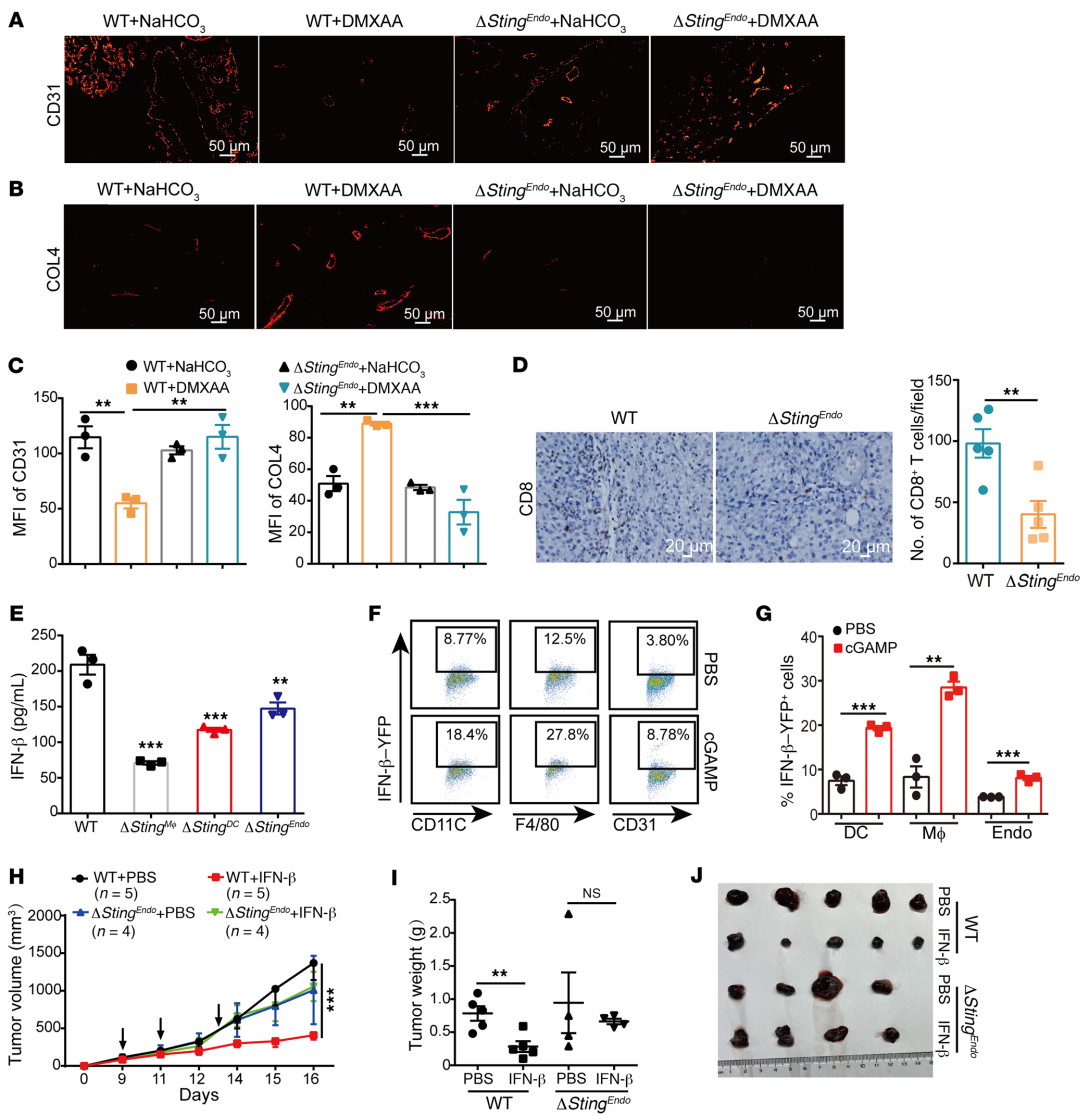
Deficiency of STING in endothelial cells impairs tumor infiltration of CD8^+^ T cells and tumor blood vessel normalization induced by STING agonists. (**A**–**C**) Representative immunofluorescent images and quantified results showing CD31 (**A**) and COL4 (**B**) expression of B16 tumor tissues from WT and *Sting^fl/fl^/Cdh5*-Cre mice after i.t. treatment of DMXAA (200 μg/mouse). Quantified results are shown in **C**. (**D**) Tumor infiltration of CD8^+^ T cells were detected by IHC after i.t. treatment of DMXAA. (**E**) Intratumoral IFN-β levels in tumor tissues from different tissue-specific *Sting*-KO mice after i.t. DMXAA treatment (200 μg/mouse) were determined by ELISA. (**F** and **G**) IFN-β–YFP^+^ expression in different cell populations within B16 tumors from IFN-β–YFP reporter mice after i.t. cGAMP (25 μg/mouse) treatment. (**H**–**J**) WT and *Sting^fl/fl^/Tek*-Cre mice were treated with i.t. injection of IFN-β (50 ng/mouse) at indicated time points (arrows), then the tumor size was recorded (**H**), weighed (**I**), and imaged (**J**) upon harvest. The number of mice used in each group was shown in the Figure. Data are represented as mean ± SEM. **P < 0.01; ***P < 0.001, by 1-way ANOVA with Šidák’s multiple comparisons test (**C** and **E**), or by 2-tailed unpaired *t* test (**D**, **G**, and **I**), or by 2-way ANOVA with Šidák’s multiple comparisons test (**H**).

As STING deficiency in endothelium abolished tumor control by intratumoral STING agonist, we next checked how the intratumoral administration of STING agonists reprogrammed tumor vasculature and the tumor microenvironment. Immunofluorescence staining on tumor tissues found that the expression level of CD31, a blood vessel marker, was significantly decreased in tumors from WT mice after the treatment of STING agonist, but not in tumors from *Sting^fl/fl^/Cdh5-Cre* mice. Meanwhile, STING agonists markedly induced intratumoral expression of COL4, a pericyte marker, which was compromised in tumors from *Sting^fl/fl^/Cdh5-Cre* mice (Figure 3, A–C). Since tumor vasculature is important for T cell infiltration, and CD8^+^ T cells are critical for the antitumor effect of STING agonist (Figure 1G), we next analyzed T cell infiltration in tumors,and found that there was a decrease in intratumoral CD3^+^ T cell and CD8^+^ T cell infiltration in *Sting^fl/fl^/Cdh5-Cre* mice upon intratumor DMXAA treatment, compared with that of WT mice (Figure 3D and Supplemental Figure 3, A and B). Adoptive transfer of CD45.1^+^ CD45.2^+^ T cells to WT and *Sting^fl/fl^/Tek-Cre* mice (CD45.2^+^ T cells) also revealed that intratumoral CD8^+^ T cell infiltration was significantly lower in endothelial *Sting*-cKO mice compared with that of WT mice upon intratumor DMXAA injection (Supplemental Figure 3C). As IFN-I signaling is also required for the antitumor effect of STING activation, we next checked intratumor IFN-β levels after treatment. Compared with that of WT mice that received STING agonist treatment, we found that IFN-β induction was significantly reduced in tumor tissues from *Sting^fl/fl^/Cdh5-Cre* mice and was even lower in tumor tissues from *Sting^fl/fl^/LysM-Cre* and *Sting^fl/fl^/Itgax-Cre* mice, indicating that all these cell types produced IFN-β upon STING activation, but none of these cKO mice completely lost IFN-β induction (Figure 3E). In line with these results, intratumoral injection of cGAMP on B16 tumor-bearing IFN-β–YFP reporter mice significantly induced IFN-β–GFP signal in endothelial cells (CD45^–^CD31^+^), as well as that in macrophages (CD45^+^F4/80^+^) and DCs (CD45^+^CD11c^+^) with even higher levels (Figure 3, F and G). In vitro stimulation of bone marrow–derived macrophages (BMDMs) and bone marrow–derived dendritic cells (BMDCs) also showed higher levels of IFN-β production than that endothelial cells upon STING activation (Supplemental Figure 3, D–F), suggesting that DCs and macrophages were the main resources of IFN-β in vivo upon STING activation. As the antitumor effect of STING agonist on *Sting^fl/fl^/LysM-Cre* and *Sting^fl/fl^/Itgax-Cre* mice was intact, despite significantly lowered intratumoral IFN-β production, though IFNAR deficiency abrogated such effect (Figure 1F), it was likely that the residual IFN-β production in these cKO mice was sufficient to induce downstream antitumor activity, which requires endothelial STING expression. Thus, we reasoned that defective downstream signaling of IFNAR, rather than reduced IFN-β induction, was responsible for the abolished antitumor effect of STING agonists on endothelial *Sting*-cKO mice. To formally test this hypothesis, we performed intratumoral injection of purified recombinant IFN-β protein into WT and *Sting^fl/fl^/Tek-Cre* mice, and found that IFN-β administration significantly inhibited tumor growth in WT mice, but not in *Sting^fl/fl^/Tek-Cre* and *Ifnar^–/–^* mice (Figure 3, H–J, and Supplemental Figure 3G). These results suggest that STING play a critical role in the regulation of endothelial normalization downstream of IFNAR activation, which is required for the antitumor activity of intratumor STING agonists.

### STING in endothelial cells is required for IFNAR downstream signaling activation.

**Figure 4:**
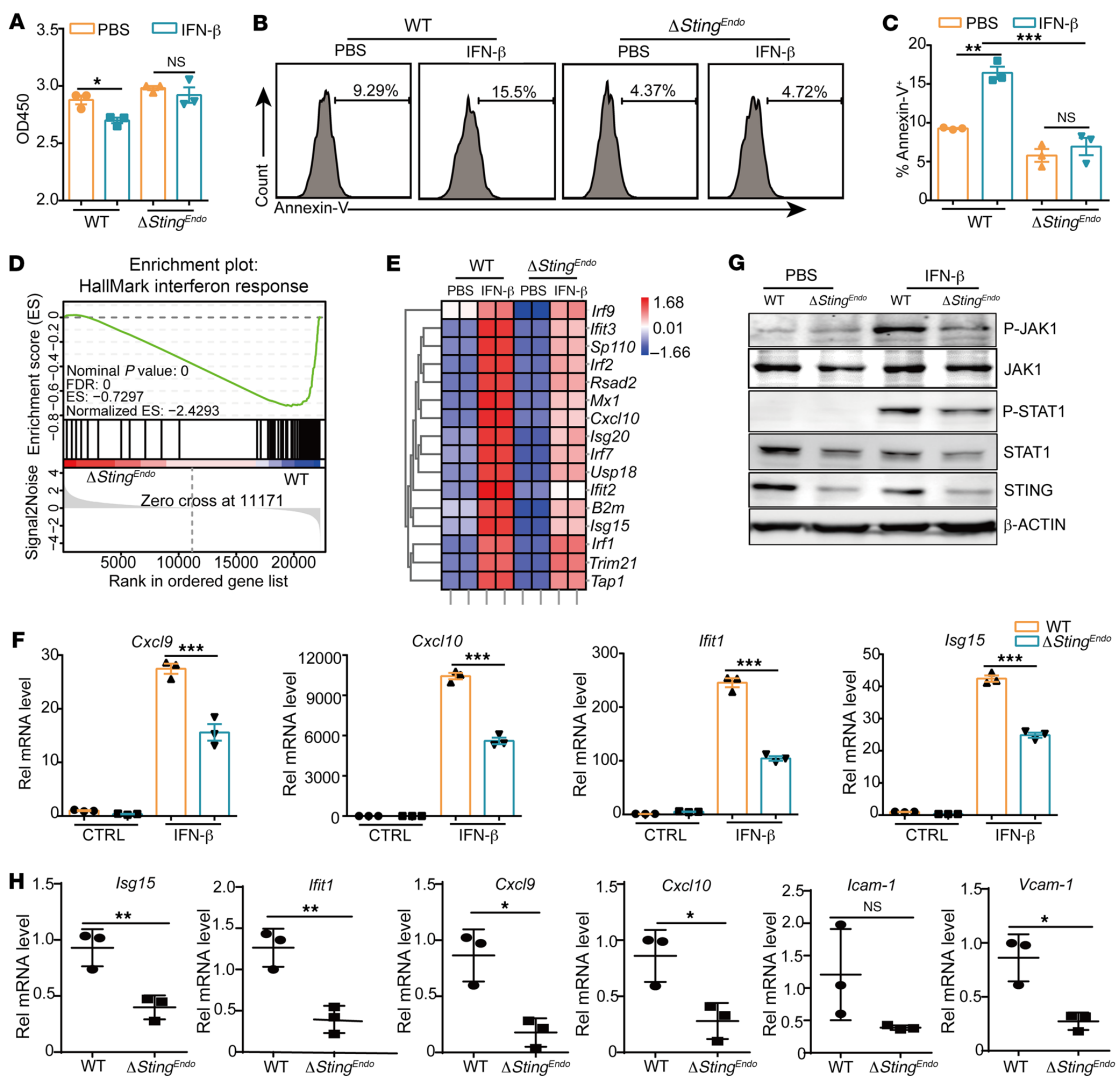
STING expression in endothelial cells is required for IFNAR downstream signaling activation. (**A**–**C**) Primary mouse endothelial cells (WT, *Sting*-KO) were stimulated with IFN-β (10 ng/mL) for 24 hours, followed by CCK8 assay (**A**), Annexin-V staining and FACS analysis (**B**). Percentage of Annexin-V^+^ cells was quantified in **C**. (**D** and **E**) RNA-seq analysis detecting IFN response and ISG gene expression in endothelial cells after IFN-β (10 ng/mL) treatment for 3 hours. (**F**) qRT-PCR analysis of *Cxcl9*, *Cxcl10*, *Ifit1*, and *Isg15* expression levels in endothelial cells after treatment with IFN-β (10 ng/mL) for 3 hours. (**G**) Western blot analysis of JAK1 and STAT1 phosphorylation levels in endothelial cells (WT, *Sting*-KO) after treatment with PBS or IFN-β (10 ng/mL) for 15 minutes. (**H**) qRT-PCR analysis of *Isg15*, *Ifit1*, *Cxcl9*, *Cxcl10*, *Icam-1*, and *Vcam-1* expression levels in WT and endothelium-specific *Sting* KO (*ΔSting^Endo^*) tumor tissues after i.t. DMXAA treatment (200 μg/mouse). Data are represented as mean ± SEM. *P < 0.05; **P < 0.01; ***P < 0.001, by 1-way ANOVA with Šidák’s multiple comparisons test (**C** and **F**), or by 2-tailed unpaired *t* test (**A** and **H**).

Previous work has shown that intratumoral IFN-β injection-mediated tumor control needed IFNAR in vascular endothelial cells (36), and our work has shown that STING in endothelial cells was critical for IFN-β–induced tumor inhibition. We also found that IFN-β treatment on endothelial cells inhibited cell proliferation and promoted apoptosis (Figure 4, A–C), which was inhibited by *Sting* deficiency, further suggesting that STING may regulate IFNAR downstream signaling in endothelial cells for antitumor activity. To test this hypothesis, we treated endothelial cells with IFN-β and performed global RNA-seq analysis to identify genes and pathways regulated by STING in endothelial cells upon IFNAR activation. As expected, gene ontology (GO) enrichment analysis showed significant enrichment for the response to IFN-β and immune-response functions (Supplemental Figure 4A). Gene set enrichment analysis (GSEA) showed *Sting* deficiency induced significant downregulation of IFN response, and the heatmap of interferon-stimulated genes (ISGs) revealed that *Sting* deficiency inhibited a variety of ISG genes upon IFN-β treatment (Figure 4, D and E). Further qPCR experiments verified that IFN-β treatment actually induced ISG expression via the STING pathway in primary mouse endothelial cells and a human umbilical vessel endothelial cell line (HUVEC), as *Sting* deficiency significantly lowered IFN-β–induced expression of ISG genes such as *Cxcl9*, *Cxcl10*, *Ifit1*, *Isg15*, *Icam-1*, *Vcam-1*, *Isg-15*, *Mx1*, and *Rsad2* (Figure 4F and Supplemental Figure 4, B–D). Moreover, IFN-β treatment on WT endothelial cells strongly induced the phosphorylation of JAK1 and STAT1, which was markedly weakened in *Sting*-KO endothelial cells (Figure 4G). Interestingly, IFN-β–induced phosphorylation of STAT1 was not downregulated in *Sting*-deficient DCs and macrophages, indicating an endothelium-specific regulation of JAK1-STAT1 signaling by STING (Supplemental Figure 4, E and F). Conversely, overexpression of *Sting* in primary endothelial cells further increased the phosphorylation levels of JAK1 and STAT1 upon IFN-β treatment (Supplemental Figure 4G). Importantly, intratumoral injection of DMXAA-induced gene expression levels of *Isg15*, *Ifit1*, *Cxcl9*, *Cxcl10*, *Icam-1*, and *Vcam-1* were all lower in *Sting^fl/fl^*/*Cdh5-Cre* mice compared with that of WT mice (Figure 4H). These results suggest that STING is required for IFNAR downstream signaling activation and ISG induction in endothelial cells, which determine proper downstream antitumor activity.

### STING interacts with JAK1 in primary endothelial cells upon IFNAR activation.

**Figure 5:**
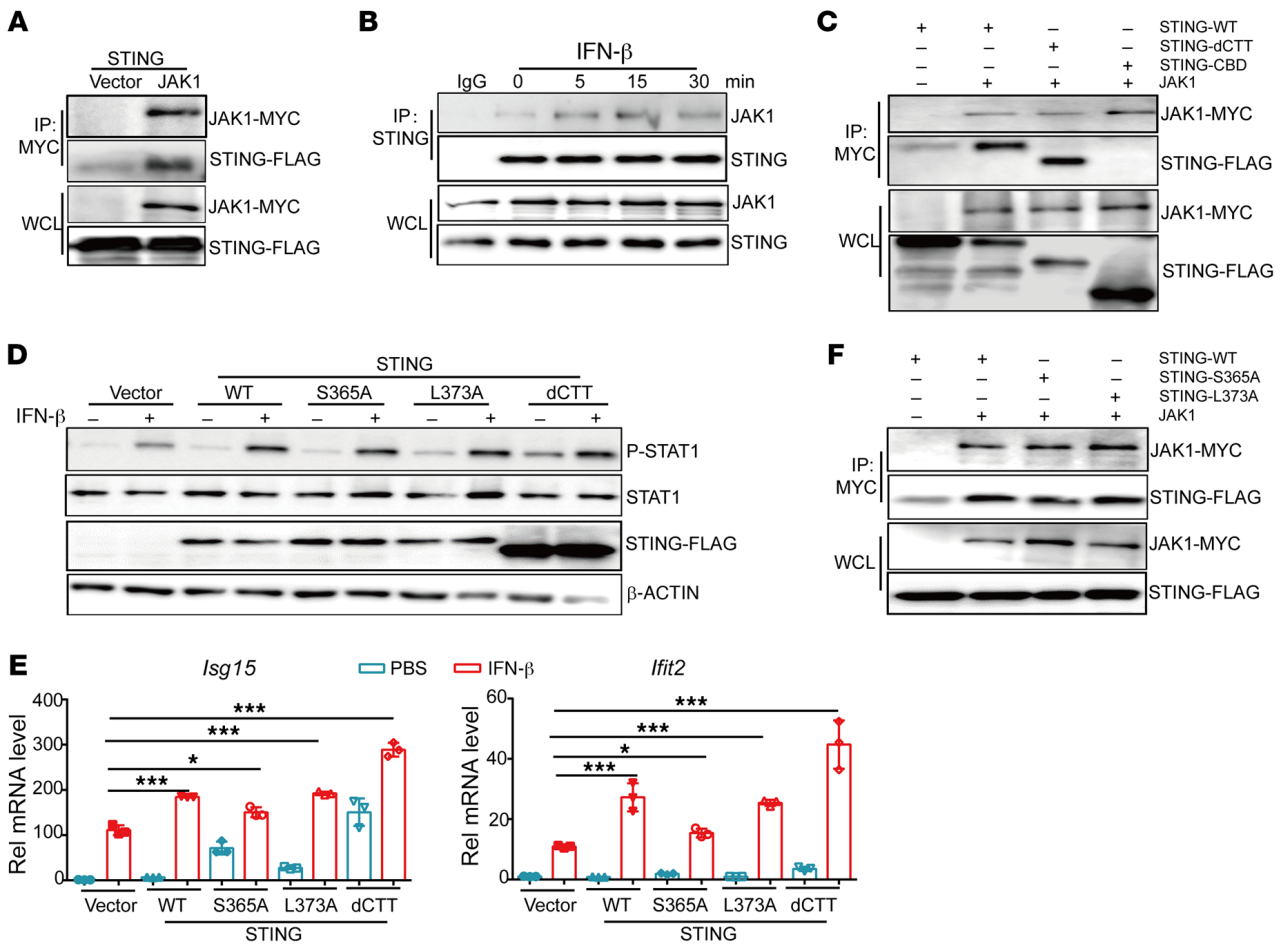
STING interacts with JAK1 in primary mouse endothelial cells upon IFN-β stimulation. (**A**) Expression vectors of STING-FLAG and JAK1-MYC were cotransfected into 293T cells and whole cell lysate (WCL) was used for immunoprecipitation (IP) with anti-MYC antibody, followed by WB with anti-FLAG or anti-MYC using immunoprecipitate or WCL (as loading control). (**B**) Primary mouse endothelial cells were treated with IFN-β (10 ng/ml), WCL was harvested at the indicated time points, and coimmunoprecipitation was performed using anti-STING antibody, followed by WB detection of JAK1 and STING in immunoprecipitate or WCL. (**C**) WT or mutant STING (dCTT, CBD) expression vectors were cotransfected with JAK1 into 293T cells, and WCL was used for IP with anti-MYC, followed by Western blot detection of STING or JAK1 in immunoprecipitate or WCL. (**D**) Primary mouse endothelial cells were infected with lentivirus expressing WT or mutant STING (S365A, L373A and dCTT), then treated with IFN-β (10 ng/mL) for 15 minutes, then WCLs were harvested for Western blot analysis of phosphorylated STAT1 (P-STAT1) and total STAT1 levels. (**E**) qPCR analysis of *Isg15* and *Ifit2* gene expression levels in primary endothelial cells shown in **D**. (**F**) WT or mutant STING (S365A, L373A) expression vectors were cotransfected with JAK1 into 293T cells, and WCL was used for IP with anti-MYC, followed by Western blot detection of STING or JAK1 in immunoprecipitate or WCL. WT, WT-STING; CBD, CDN binding domain; S365A, serine 365-to-alanine; L373A, leucine 373-to-alanine; dCTT, deficiency of CTT domain. Data in (**E**) are represented as mean ± SEM. *P < 0.05; ***P < 0.001, by 1-way ANOVA with Šidák’s multiple comparisons test.

Previous studies have identified multiple STING interaction proteins, among which JAK1 interaction with STING has not been studied. To further explore the underlying mechanism responsible for impaired JAK1 phosphorylation in *Sting* KO endothelium after IFN-β stimulation, we tested the interaction between STING and JAK1 and found that STING could interact with JAK1 (Figure 5A) and that the interaction was enhanced and peaked 15 minutes after IFN-β stimulation (Figure 5B), which correlates with the dynamics of JAK1 phosphorylation level (Supplemental Figure 5A). To further explore which domain of STING was involved in the interaction of JAK1 and STING, we constructed STING truncation mutants of dCTT (deficiency of CTT for TBK1 binding) and CBD (CBD only). Results showed that the lack of a CTT domain did not impair the interaction of STING and JAK1, while the CBD domain only could not interact with JAK1 (Figure 5C). Moreover, overexpression of WT, S365A, L373A (mutation of 2 sites required for TBK1 and IRF3 binding), and dCTT mutants into primary endothelial cells all enhanced phosphorylation levels of STAT1 and ISG expression levels induced by IFN-β treatment (Figure 5, D and E, and Supplemental Figure 5B). Consistently, JAK1 interaction with WT STING was comparable to that of STING-S365A and STING-L373A in 293T cells (Figure 5F). These results suggest that STING can interact with JAK1 in primary endothelial cells to promote STAT1 phosphorylation and downstream ISG expression, independent of its CTT domain.

### STING C91A mutation disrupts its interaction with JAK1 and impairs JAK1 activation.

**Figure 6:**
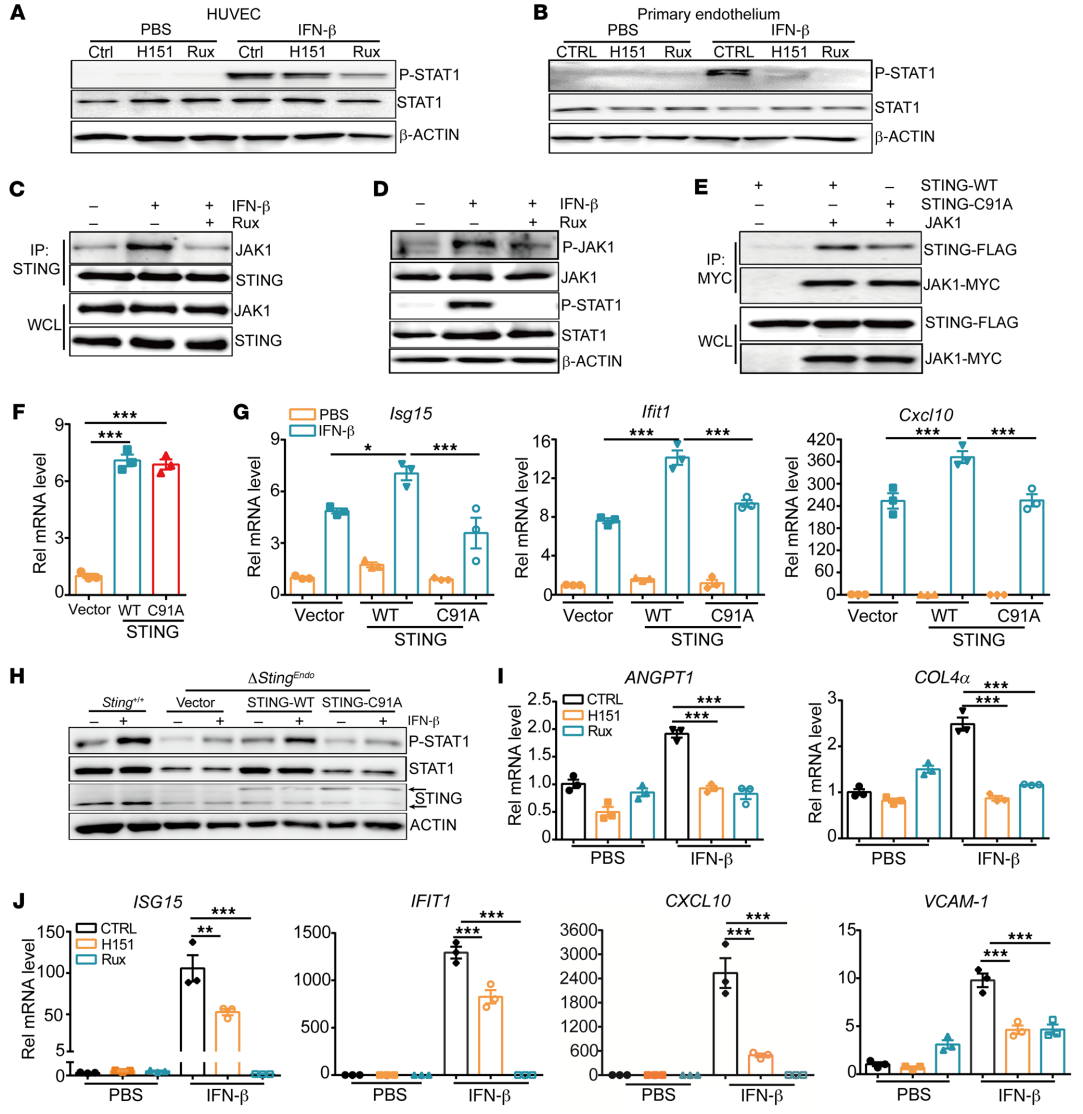
STING C91A mutation disrupts its interaction with JAK1 and impairs JAK1 activation upon IFN-β stimulation. (**A** and **B**) HUVEC cells (**A**) or primary mouse endothelial cells (**B**) were stimulated with IFN-β (10 ng/mL) for 15 minutes with or without pretreatment with H151 (10 μg/mL) or JAK1 inhibitor (ruxolitinib, rux, 5μM), then the WCL was used for Western blot analysis of phosphorylation levels of STAT1. (**C**) Co-IP assay was performed to detect the interaction between STING and JAK1 in primary mouse endothelial cells stimulated with IFN-β (10 ng/mL) for 15 minutes, with or without pretreatment with ruxolitinib (5 μM) for 30 minutes. (**D**) WB analysis of phosphorylation level of JAK1 and STAT1 in mouse endothelial cells stimulated with IFN-β (10 ng/mL) for 15 minutes, with or without pretreatment with ruxolitinib. (**E**) Co-IP assay was performed to detect the interaction between JAK1 and WT or C91A mutant STING after coexpression in 293T cells. (**F** and **G**) Primary mouse endothelial cells were infected with lentivirus expressing WT or C91A mutant STING, followed by qPCR analysis of *Sting* gene (**F**) and *Isg15*, *Ifit1*, and *Cxcl10* expression levels after IFN-β (10 ng/mL) stimulation for 3 hours (**G**). (**H**) Western blot analysis of phosphorylation level of STAT1 IFN-β–stimulated (10 ng/mL, for 15 minutes) primary endothelial cells (WT or *Sting*-KO cells with reexpression of WT or C91A mutant STING. The upper band of STING blot indicated the exogenous WT and C91A STING protein, while the lower band indicated the endogenous STING protein. (**I**–**J**) qPCR analysis of the expression of *ANGPT1*, *COL4a*, and other ISG genes (*ISG15*, *IFIT1*, *CXCL10*, and *VCAM-1*) in HUVEC cells stimulated with IFN-β (10 ng/ mL) for 3 hours, with pretreatment of H151 (10 μg/mL) or Ruxolitinib (5 μM) for 30 minutes. Data are represented as mean ± SEM. *P < 0.05; **P < 0.01; ***P < 0.001, by 1-way ANOVA with Šidák’s multiple comparisons test (**F**, **G**, **I**, and **J**).

To examine whether STING and JAK1 activation is required for STING and JAK1 interaction, we next used H151, a STING antagonist, and ruxolitinib, a JAK1 antagonist (37, 38), to treat HUVEC cells and primary mouse endothelial cells. Both antagonists weakened the phosphorylation of STAT1 induced by IFN-β stimulation (Figure 6, A and B). Moreover, ruxolitinib treatment decreased the interaction of JAK1 and STING along with inhibiting the phosphorylation of JAK1 and STAT1 (Figure 6, C and D). As H151 exerts its inhibitory activity via blocking STING palmitoylation on Cysteine 91 and its clustering (37), we next tested if palmitoylation was required for STING interaction with JAK1. Indeed, the STING C91A (cysteine to alanine) mutation attenuated the interaction between STING and JAK1 (Figure 6E). In support of this, pretreatment of primary endothelial cells with a protein palmitoylation inhibitor, 2BP, also downregulated JAK1 and STAT1 phosphorylation and ISG expression levels by IFN-β stimulation (Supplemental Figure 6, A and B). Overexpression of WT STING in primary endothelial cells promoted IFN-β–induced expression of ISGs including *Isg15*, *Ifit1*, and *Cxcl10*, but C91A significantly weakened such induction (Figure 6, F and G). In line with this, overexpression of WT-STING, but not C91A mutant, restored IFN-β–induced STAT1 phosphorylation level in *Sting*-KO endothelial cells (Figure 6H). Importantly, H151 and ruxolitinib treatment on endothelial cells both inhibited IFN-β–induced expression of genes related to vasculature normalization, such as *ANGPT1* and *COL4a*, in addition to classical ISGs (*ISG15*, *IFIT1*, *CXCL10*, *VCAM-1*, and *IFIT2*) (Figure 6, I and J, and Supplemental Figure 6C). These results suggest that STING palmitoylation and JAK1 interaction may facilitate JAK1/STAT1 activation and promote downstream ISG and vasculature normalization-related gene expression in endothelial cells, which play a pivotal role in immune cell infiltration and ensuing antitumor immune responses.

### Endothelial STING expression positively correlates with endothelial JAK1-STAT1 signaling and antitumor immunity.

**Figure 7:**
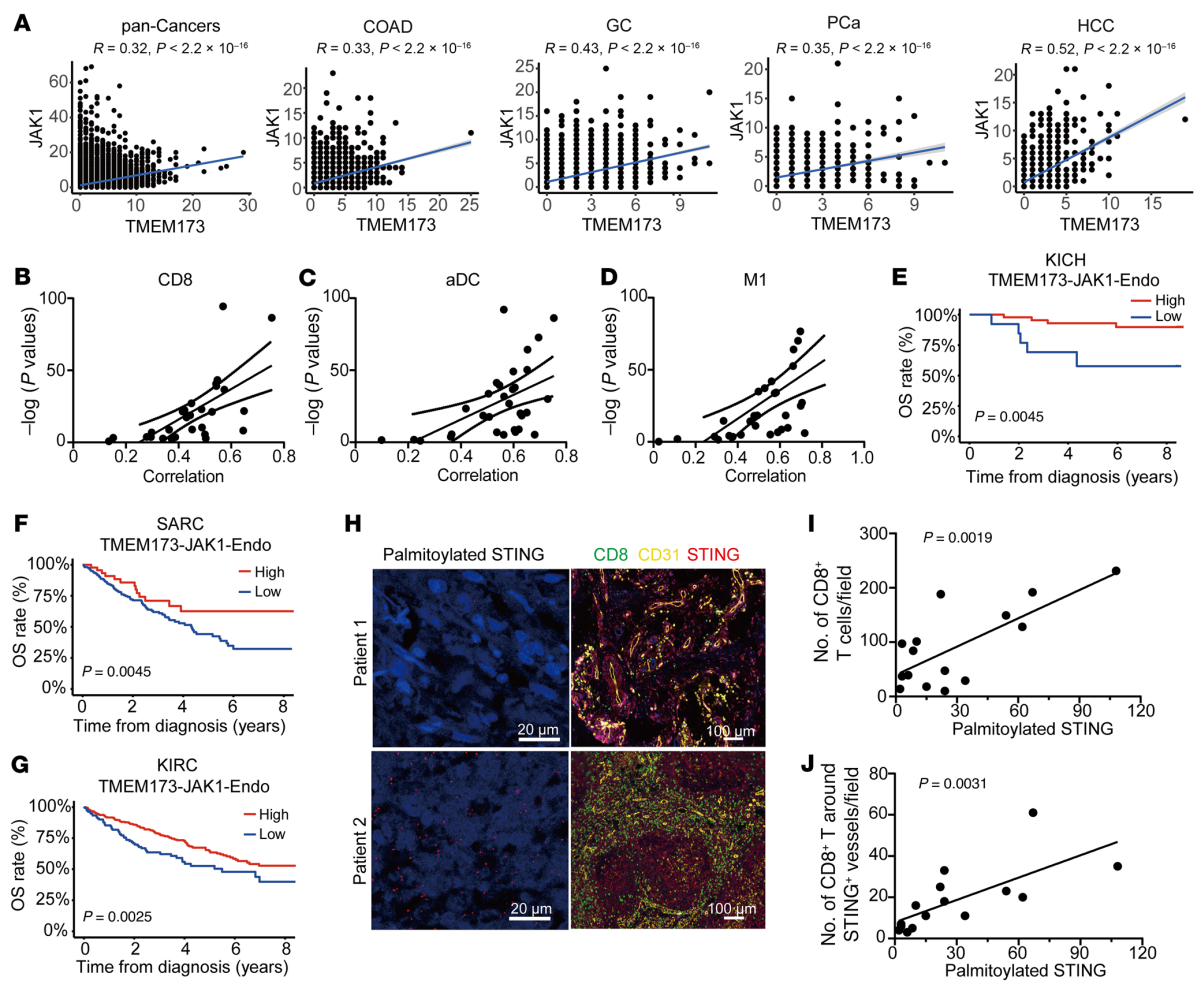
Endothelial STING expression positively correlates with endothelial JAK1-STAT1 signaling and antitumor immunity. (**A**) Correlation between endothelial STING expression and endothelial JAK1 expression in pancancers and COAD, GC, PCa, and HCC cancers. (**B**–**D**) Correlation between endothelial STING-JAK1 expression and intratumoral CD8^+^ T cells, activated DCs, and M1 Macrophages. (**E**–**G**) Kaplan-Meier survival curves of KICH, KIRC, and SARC patients stratified by endothelial STING-JAK1 expression levels. (**H**) STING palmitoylation and CD31, STING, and CD8 expression in melanoma tissues were detected by PLA assay and multiplex immunofluorescence, respectively. (**I** and **J**) Correlation between STING palmitoylation and CD8^+^ T cell infiltration (**I**), CD8^+^ T cell infiltration around STING-positive vessels (**J**), *n* = 15. COAD, colon adenocarcinoma; GC, gastric carcinoma; PCa, prostate cancer; HCC, hepatocellular carcinoma; KICH, kidney chromophobe; KIRC, kidney renal clear cell carcinoma; SARC, soft tissue sarcoma. *R* and *P* values by Pearson’s correlation test (**A**–**D**, **I**, and **J**). *P* values by the log-rank test (**E**–**G**).

To validate the correlation between STING and JAK1 in vascular endothelial cells in clinical databases, we performed a correlation study on endothelial signaling with JAK1-STAT1 signaling using the published datasets from tissues from patients with cancer. The results showed that there was a positive correlation between STING and JAK1 or STAT1 expression in vascular endothelial cells in pancancers and a variety of cancer types, including colorectal adenocarcinoma (COAD), gastric carcinoma (GC), prostate cancer (PCa), and hepatocellular carcinoma (HCC) (Figure 7A and Supplemental Figure 7A). Using the published single-cell datasets, we also observed the positive correlation of STING and JAK1 expression in vascular endothelial cells from patients with PCa, COAD, GC, primary liver cancer (PLC), glioblastoma (GM), glioblastoma multiforme (GBM), and upper tract urothelial carcinoma (UTUC) (Supplemental Figure 7B). In addition, the expression of STING and JAK1 in vascular endothelial cells was positively associated with the infiltration of CD8^+^ T cells, activated DCs, and M1 macrophages in the tumor microenvironment of pancancers (Figure 7, B–D). Moreover, Kaplan-Meier survival analysis showed that higher expression of STING/JAK1 or STING/ISGs in vascular endothelial cells was associated with better overall survival rate in patients with kidney chromophobe (KICH), kidney renal clear cell carcinoma (KIRC), and soft tissue sarcoma (SARC) (Figure 7, E–G, and Supplemental Figure 7C). These results suggest that STING was positively associated with JAK1 expression and anticancer immunity in patients with cancer.

Furthermore, to investigate the clinical relevance of STING palmitoylation and tumor immunity, we examined the level of STING palmitoylation by a modified acyl-biotinyl exchange (ABE) assay integrated with proximity ligation assay (PLA), and the expression of CD31, STING, and CD8^+^ T cell expression by multiplex immunofluorescence staining in tumor tissue from patients with melanoma. We found that the level of STING palmitoylation was positively correlated with the level of CD8^+^ T cell infiltration in tumor tissues, as well as the level of CD8^+^ T cells around STING-positive blood vessels (Figure 7, H–J).

## Discussion

Our present findings demonstrate the importance of endothelium-specific STING function in mediating intratumor STING agonist–induced tumor inhibition. Unexpectedly, *Sting* knockout in macrophages or DCs had no effect on intratumoral STING agonist–induced tumor control in vivo, while *Sting* knockout in endothelium almost completely abolished this tumor inhibitory effect. This defect was not due to impaired intratumoral IFN-β production, as even lower levels of IFN-β production in tumors of DC- or macrophage-specific *Sting*-KO mice still had an intact tumor inhibition effect. Strikingly, the impaired JAK1-STAT1 activation and downstream gene expression after IFNAR activation in *Sting^–/–^* endothelium are responsible for the lost tumor inhibition effect of the STING agonist. Upon IFN-β stimulation, STING binds to phosphorylated JAK1 and facilitates JAK1-STAT1 pathway activation and downstream ISG induction. Moreover, STING interacts with and activates JAK1 depending on the palmitoylation of STING at the C91 site, but not the TBK1/IRF3-interacting CTT domain. Overall, our work identifies an endothelium-specific function of STING in mediating intratumor agonist-induced antitumor activity and reveals a new mechanism by which STING facilitates JAK1-STAT1 signaling activation for tumor vascular normalization upon IFNAR activation.

Tumor intrinsic STING signal commonly plays an essential role in preventing cancer development, and tumors often reduce STING expression levels to escape immune surveillance (39). However, previous studies have demonstrated that intratumoral STING agonists often override the need for STING expression in tumor cells for antitumor activity (35). Consistently, we found that tumor-intrinsic STING expression was dispensable for intratumor STING agonist–induced tumor control. In contrast, activation of STING signaling in antigen-presenting cell types (e.g., macrophages and DCs) was found to be critical for inducing a tumor-specific adaptive immune response induced by irradiation therapy or STING agonist–adjuvanted cancer vaccine (25, 40). Notably, we identified that STING signaling in macrophages and DCs were not essential for intratumoral STING agonist–induced MC38 or B16 mouse tumor control. We reasoned that irradiation-induced tumor DNA release or cancer vaccine mainly targeted DCs for their activation, while intratumor STING agonists directly acted on tumor endothelium for vasculature normalization to facilitate T cell infiltration.

Previous studies suggested that endothelial cells were the main cell source for intratumor IFN-β production and secretion upon stimulation with STING agonists and that IFN-β directly acted on endothelial cells in an autocrine or paracrine manner (27). However, we found a relatively limited amount of IFN-β production in the endothelium after STING agonist stimulation both in vitro and in vivo when compared with DCs and macrophages. Most importantly, reduced intratumor IFN-β production did not impair the antitumor activity of STING agonists on DC- or macrophage-specific *Sting*-KO mice, suggesting that IFN-β was derived from multiple sources, and residual IFN-β was sufficient for activating endothelium for STING agonist-induced antitumor activity. However, the residual IFN-β in endothelium-specific *Sting*-KO mice is not sufficient for the antitumor activity. Thus, STING function downstream of IFN-β stimulation in endothelial cells was essential for the antitumor activity. In line with our findings, a recent study showed that brain endothelial cells are the primary target cells and the mediator responsible for excessive intracerebral IFN-α–induced neurotoxicity in a mouse model for Aicardi-Goutières syndrome (AGS) (41). Together, we propose that STING not only senses agonist binding and activates TBK1/IRF3 for IFN-I gene induction, but also plays an essential role in proper JAK1-STAT1 signaling transduction in endothelial cells upon IFNAR stimulation. The latter has not been previously reported and may have important implications for future design of STING agonist–based cancer therapy.

Another key observation of the present study is that STING interacts with JAK1 to regulate JAK1-STAT1 signal transmission in endothelium upon IFN-β stimulation. It is well known that STING was activated by cGAMP binding and in turn mediated TBK1, IRF3, and NF-κB activation (42). One study showed that STING could recruit STAT6 to the endoplasmic reticulum, leading to STAT6 phosphorylation by TBK1 independent of JAKs (43). However, in our study, we found that the CTT domain of STING, which was responsible for TBK1 and IRF3 recruitment and activation, was not essential for the JAK1 and STING interaction in endothelial cells. Unlike STING interaction with TBK1 and IRF3, STING interacted with JAK1 via the N-terminal and CBD. Previous studies have shown that N terminal and CBD domain and STING-C88/C91 palmitoylation were essential for STING binding with other molecules (44–47). Consistently, the palmitoylation of STING at the C91 site was also critical for STING interaction with JAK1 in endothelial cells in the current study. Thus, it seemed that STING relied on its C terminal domain for TBK1/IRF3 activation, and used its N-terminal domain and palmitoylation to promote JAK1-STAT1 signaling.

Our results showed that IFN-β treatment with endothelial cells could prevent its proliferation and induce apoptosis, leading to a decrease of vessel density. Moreover, the STING signaling in the endothelium could influence vessel pericyte coverage, which involved vessel normalization, which promotes T cell infiltration to remodel the tumor environment (29, 48). In the current study, we found that CD8^+^ T cells were recruited to the tumor environment within tumor tissues via STING signaling activation in endothelium. Depletion of CD8^+^ T cells, but not CD4^+^ T cells, abolished the STING agonist–mediated antitumor effect. This was consistent with the findings from 2 recent studies reporting nanoformulated STING agonists for tumor endothelium delivery (49, 50). Importantly, STING expression in the endothelium was also found critical for such nanoformulated agonist-induced vasculature normalization and CD8^+^ T cell infiltration, echoing our findings using intratumoral administration of STING agonist for cancer treatment.

In conclusion, our results demonstrated an essential and unique role of STING in the endothelium, mediating intratumoral STING activation–induced antitumor activity. STING was not only required for agonist binding, TBK1/IRF3 activation and IFN-α/β gene induction, but also needed for proper JAK1-STAT1 activation and ISG induction downstream of IFNAR activation. STING signal in the endothelium acted both upstream and downstream of IFN-I to promote vessel normalization and facilitate CD8^+^ T cell infiltration, which was critical for STING agonist–induced antitumor immunity. Importantly, we demonstrated a positive correlation between endothelial STING expression and STING palmitoylation with CD8^+^ T cell infiltration in tumor tissues of patients with cancer, further corroborating the clinical relevance of our findings.

In summary, our findings highlight a previously unrecognized function of STING in regulating JAK1/STAT activation downstream of IFNAR signaling in endothelial cells and provide a new critical insight for future design and clinical application of STING agonists for cancer therapy.

### Limitations of the study.

Although we used a series of tissue-specific knockout mice to determine the endothelium-specific function of STING in antitumor immunity, we mainly relied on intratumor injection of STING agonists as the treatment module. It is possible that different administration routes of STING agonists may activate different cell types to promote antitumor immunity. The conditional knockout mouse models would be a good tool to test such a possibility.

Another limitation of the study is how STING palmitoylation was induced upon IFN-β stimulation, and whether such modification promotes STING translocation to interact with JAK1 remains unknown. In this study, we focused on STING agonist–induced antitumor immunity for cancer treatment. It would also be imperative to test the pathophysiological role of JAK1-STING interaction in other human diseases related to dysregulated IFN-I signaling.

## Methods

### Sex as a biological variable.

In this study, sex was not considered as a biological variable in the animal experiments. In human tumor tissue samples studies, sex was not considered as a biological variable.

### Construction of tissue-specific Sting knockout mice.

C57BL/6J female mice 6-to-8 weeks of age were obtained from Viral River Laboratory, Beijing. *Itgax*-Cre, *Tek*-Cre, and *Ifnar*^–/–^ mice were obtained from Jackson Laboratory, and *Cdh5*-CreERT2 mice were provided by Leming Zheng at Peking University Health Science Center (Beijing, China). *Tmem173*^fl/fl^ (*Sting*^fl/fl^) mice were purchased from Shanghai Model Organisms Center Inc. (Shanghai, China). *Sting*^fl/fl^ mice were crossed with transgenic mice expressing specific Cre-recombinase to generate *Sting*^fl/fl^/*Itgax-*Cre, *Sting*^fl/fl^/*LysM*-Cre, *Sting*^fl/fl^/*Tek*-Cre, and *Sting*^fl/fl^/*Cdh5*-Cre mice, which specifically knockout *Sting* in DCs, macrophages, and endothelium, respectively. And the littermate *Sting*^fl/fl^ mice were used as WT control. Genotyping of the mice was performed by PCR. All mice were maintained under specific pathogen-free conditions.

### Cell lines.

The B16 (C57BL/6J mouse melanoma) cells were purchased from ATCC. MC38 (C57BL/6J mouse colon adenocarcinoma) cells were provided by Yang Xuanming (Shanghai Jiaotong University, Shanghai, China). HUVECs were provided by Huanhuan He (The Fifth Affiliated Hospital, Sun Yat-sen University, Zhuhai, China). B16-OVA cells were constructed by stably expressing OVA cDNA on B16 cells. *Sting*-deficient B16 and MC38 cells were constructed through the CRISPR/Cas9 technology using the guide RNA with sequences 5′-GTACCTTGGTAGACAATGAGG-3′. All cells were maintained with DMEM (Invitrogen) or RPMI-1640 (Invitrogen) medium supplemented with 10% FBS (TransGen Biotech) and 1% penicillin-streptomycin (Gibco) in a humidified atmosphere at 37°C and 5% CO_2_. All cell lines were routinely tested and determined to be mycoplasma free.

### Construction of STING truncation and mutants.

Full-length STING with FLAG tag and STING truncation mutants with CBD (amino acids 138–344), or the deficiency of CTT (dCTT, amino acids 1–330) were expressed in HEK 293T cells together with JAK1-MYC vector. STING mutant at site C91A, S365A, and L373A were also expressed in HEK 293T cells with JAK1-MYC. Then, protein sample were prepared and quantified for Western blotting. These vectors were cotransfected with the pspax2 and pMD2.G packaging plasmids, and the supernatant was harvested 48 hours after transfection. The supernatant was centrifuged at 48,000*g* for virus enrichment and the virus was used to infect primary vascular endothelial cells to construct STING-overexpressing cells.

### Generation of BMDC, BMDM, and endothelium.

For BMDC or BMDM induction, single-cell suspensions of mouse bone marrow cells were cultured in RPMI-1640 medium containing 10% FBS, supplemented with 20 ng/mL GM-CSF (PeproTech, Cat 315-03) and 20 ng/mL IL-4 (PeproTech, Cat 214-14) or 20 ng/mL M-CSF (PeproTech, Cat 315-02) for 5 days. The culture medium was refreshed every 2 days.

Endothelium was isolated from macrovessels that were minced finely, adhered to a 6-well plate, and cultured with DMEM supplement with 20% FBS and ECGS (Merck, Cat 02-102). When cells emerged, they were digested with trypsin, and continued to culture.

### Tumor models and treatment regimens.

B16 tumor cells (2 × 10^5^), B16-OVA tumor cells (2 × 10^5^), and MC38 tumor cells (1 × 10^6^) were subcutaneously implanted into WT mice, specific *Sting-KO* mice, and *Ifnar-KO* mice. When tumors reached 100–200mm^3^ in volume, the mice were randomly divided into groups and the operators were blinded to the group assignments. DMXAA (APExBIO, Cat A8233, 200 μg/mouse) resuspended in 5% of NaHCO_3_ or cGAMP (APExBIO, Cat B8362, 25 μg/mouse) formulated in PBS and vehicle control were injected by intratumorally (25 μL system). DMXAA was injected once and cGAMP was injected once every 2 days for a total of 2 injections. IFN-β (R&D Systems, Cat 8234-MB, 50 ng/mouse) dissolved in PBS was intratumorally injected once every 2 days, for a total of 3 injections. Anti-IFN-γ (BioXCell, Cat BE0055, 100 μg/mouse), anti-CD4 (BioXCell, Cat BE0003-1, 100 μg/mouse) and anti-CD8 (BioXCell, Cat BE0061, 100 μg/mouse) blocking antibodies were administrated by intraperitoneal injection, once every 3 days. Tumor volumes were measured every 2 days using calipers, and the tumor volume was calculated with the formula V = (length × width^2^)/2. Rechallenge experiments were conducted on the opposite flanks of the mice who achieved complete tumor regression for several weeks after treatment, and naive mice were used as controls.

### Real-time PCR analysis.

Total RNA was isolated using RNA Extraction Kit (Promega, Cat LS1040) according to the manufacturer’s instructions. RNA was reverse transcribed using the PrimeScript Reverse Transcriptase Reagent Kit (Takara, Cat RR036A) and Real-time PCR was performed using the SYBR Premix Kit (Genstar, Cat A301). Results were analyzed using the Bio-Rad CFX96 thermal cycler. The gene fold changes were calculated by using a ΔΔCT method and normalized to the expression of β-actin.

### Western blot, coimmunoprecipitation, immunofluorescence, and IHC.

For Western blot, endothelium was treated with IFN-β (R&D Systems, Cat 8234-MB, 10 ng/mL) for 15 minutes, and protein samples were prepared and quantified, then loaded on a SDS-PAGE gels for separation and transferred to a PVDF membrane. The following antibodies were used for Western blot analyses purchased from Cell Signaling Technology: STING (CST, Cat 13647S), STAT1 (CST, Cat 14994S), P-STAT1(CST, Cat 9167S), JAK1 (CST, Cat 50996S), and P-JAK1 (CST, Cat 74129S); Protein bands were visualized by chemiluminescence using an ECL detection kit.

For coimmunoprecipitation, endothelium treated with IFN-β (R&D Systems, Cat 8234-MB, 10 ng/mL) or not were collected, extracted, and quantified; Protein lysates were incubated with anti-STING antibody (CST, Cat 13647S, 2 μL) and rotated over night; Protein A beads (Thermo Scientific, Cat 20333, 10 μL) were added and incubated for another 2 hours. After washing 4 times, proteins were used to detect JAK1 levels for Western blot. In 293T cells, WT-STING-FLAG, FLAG-STING truncations and mutants, and JAK1-MYC were transfered to 293T cells; 48 hours later, cells were collected, extracted, and quantified, and 200 μg protein was used to culture with anti-MYC-beads (Bimake Inc., Cat B26302) overnight. After 4 washes, proteins were used to detect STING-FLAG levels for Western blot.

For immunofluorescence and IHC, the paraffin sections were deparaffinized in xylene, rehydrated, and incubated in serial ethanol baths (100%–75%, 5 minutes per bath). Epitope retrieval was performed through incubation in 10 mM EDTA buffer (pH = 8.0) with high fire treatment for 10 minutes and low fire treatment for 10 minutes. The tissue slides were then blocked with 5% BSA and incubated overnight at 4°C with anti-CD8 (CST, Cat 98941S) or anti-CD3 (Abcam, Cat ab16669) primary antibodies (for IHC) and anti-CD31 (Abcam, Cat ab182981) or anti-COL4 (Merck, Cat ab236640) primary antibodies (for immunofluorescence). After washed with PBS, the slides were incubated for 1 hour at room temperature with a secondary antibody, and the signal was subsequently detected by the chromogenic substrate. Multiplex immunofluorescence procedure was used to detect CD8, STING, and CD31 expression in human melanoma tumors. Antibodies used were anti-human CD8 (ZSGB-BIO, Cat ZA-0508), anti-human STING (Invitrogen, Cat MA5-26030), and anti-human CD31 (Abcam, Cat ab28364). All 3 antibodies were conjugated to fluorophores and diluted to optimized working concentrations with 5% BSA.

### STING palmitoylation detection in tumor tissue from patients with melanoma.

Tumor samples were obtained from 15 patients with melanoma from the Sun Yat-sen University Cancer Center (SYSUCC; Guangzhou, China). The palmitoylation of STING in tumor tissue sections were detected in accordance with the method described in the literature (51). Briefly, tumor tissue sections were heated at 65°C for 1–2 hours, deparaffinized in xylene replacement, and rehydrated by incubation in graded alcohol. The antigen retrieval was carried out using EDTA (ZSGB-Bio) with a pH = 8.0 and the antigen retrieval solution was boiled for 20 minutes. After washing the tumor tissue 3 times with ABE buffer (150 mM NaCl [Sigma-Aldrich], 50 mM HEPES pH 7.4 [Sigma-Aldrich], 10 mM EDTA [Fdbio Science], 0.2% Triton X-100 [Sigma-Aldrich]), NEM buffer (50 mM NEM [Sigma-Aldrich],150 mM NaCl, 50 mM HEPES pH 7.4, 10 mM EDTA, 0.2% Triton X-100) was added and incubated at 37°C for 30 minutes. Samples were washed 3 times with ABE buffer, to which 4 mM iodoacetamide (Sigma-Aldrich, Cat 16125, diluted in ABE buffer) was added and incubated overnight at 4°C. The next day, the samples were further incubated with 4 mM iodoacetamide for 30 minutes at room temperature. The samples were washed 3 times with ABE buffer and incubated for 1 hour with 0.7 M hydroxylamine (Sigma-Aldrich, Cat 431362, diluted in ABE buffer). The samples were then washed once with ABE buffer and incubated with 0.7 M hydroxylamine for 1 hour at room temperature again. Sections were incubated with 10 μM iodoacetamidealkyne (MCE, Cat HY-136205, in DPBS dilution) for 1 hour at room temperature and washed once with ABE buffer and 3 times with DPBS. After 3 washes with DPBS, the buffer complex (25 μM OG488 azide (Click Chemistry Tools, Cat 1264-1), 1 mM CuSO4 (Merck, Cat 7758-98-7), 1 mM tris (2-carboxyethyl) phosphine (TCEP, Merck, Cat 51805-45-9), in DPBS dilution) was added to the samples for 45–60 minutes at room temperature, protected from light. After blocking with Duolink blocking solution for 1 hour at 37°C, the appropriate STING antibody (Invitrogen, Cat MA5-26030) and fluorescein/OG 488 (Life Technologies, Cat A-889) were added and incubated for overnight. The next day, sections were washed and the Duolink in situ proximity ligation assay (PLA) was performed according to the manufacturer’s protocol. Briefly, sections were washed and incubated for 1 hour at 37°C with Duolink PLA probes MINUS and PLUS (Merck, Cat DUO92002 and DUO92004). A Duolink in situ detection kit (Merck, Cat DUO92008) was used to ligate and amplify the signal, and nuclei were stained with DAPI (Merck, Cat DUO82040). Finally, images were captured using confocal scanning fluorescence microscopy (ZEISS, LSM880), with red spots indicating palmitoylated STING signal.

### Correlation analysis between STAT1/JAK1 and TMEM173 expression in endothelial cells.

To explore the correlation between STAT1/JAK1 and TMEM173 expression in endothelial cells, we obtained single-cell sequencing expression matrix data from several published datasets for different cancers (52, 53), including non-small cell lung carcinoma (NSCLC-Tumor: PRJNA634159, GSE148071, GSE127465, GSE189357, GSE207422, UKIM-V, UKIM-V-2), lung adenocarcinoma (LUAD:HRA000154, PRJCA001731), prostate cancer (PCa-Tumor: GSE176031, HRA000823), pancreatic ductal adenocarcinoma (PDAC: GSE155698, GSE212966, OEP003254), primary liver cancer (PLC-Tumor: HRA001748), skull base chordoma (SBC: GSE202371), secondary liver cancer (SLC-Tumor: HRA001748), upper tract urothelial carcinoma (UTUC-Tumor:HRA001867), gastric carcinoma (GC: PRJNA776683, GSE206785), glioblastoma (GM-Tumor: GSE202371), glioblastoma multiform (GBM-Tumor: GSE162631), colorectal cancer (CRC-Tumor: GSE178341, HRA000963). Quality control filtering, variable gene selection, dimensionality reduction, and cell clustering for cells were performed using the Seurat package1 (version 4.4.0). All analysis packages were run in R software (version 4.3.2), with default settings unless otherwise stated. For each sample, we removed cells of low quality (UMI < 1,000, gene number < 500, and mitochondrial genome fragments > 0.2) and genes with low abundance (0.1% of all cells). The remaining cells were then normalized using the NormalizedData function, followed by scaling to regress the UMIs using the ScaleData function (negative binomial model). Principal component analysis (PCA) was performed using the “RunPCA” function based on the top 2,000 highly variable genes (HVGs) identified using “FindVariableGenes” (“mean.cutoff” ≥ 0.1, “dispersion.cutoff” ≥ 0.5). The single cell variational inference (scVI)18 algorithm was then used to correct for batch effects across different tumour types, with batch variation set to sample. We used perplexity 1 for “FindClusters” to identify large cell clusters and visualized cells using Uniform Manifold Approximation and Projection (UMAP). Subsequently, differential expression markers or genes were identified using the Wilcoxon test implemented in the FindAllMarkers function, which was considered significant with an average log_2_ (fold-change) of at least 0.25 and a Bonferroni adjusted *P* value less than 0.05. Endothelial cell clusters were pinpointed by high expression levels of VWF, KDR, ENG, and PECAM1. Finally, Pearson correlation was used to assess the relationship between STAT1/JAK1 and TMEM173 within these cells across different cancer types.

We also used the previously published pancancer single cell data on tumor endothelial cells (TECs) to assess the correlation of TMEM173 with the JAK1-STAT1 axis (54). The UMI counts of genes were adopted. The following 11 cancer types were analyzed: breast cancer, cervical cancer, esophageal squamous cell carcinoma, gastric cancer, hepatocellular carcinoma, intrahepatic cholangiocarcinoma, lung adenocarcinoma, prostate cancer, pancreatic ductal adenocarcinoma, ovarian cancer, and head and neck squamous cell carcinoma.

To assess the correlation of TMEM173^+^ TECs with other components in the TME, we generated the scores of TMEM173^+^ TECs and common components in the TME based on the TCGA pancancer RNA-seq data using the single-sample gene-set enrichment analysis (ssGSEA) method. Markers of common components in the TME are derived from the XCell package (55). Furthermore, we also assessed the impact of TMEM173^+^ TECs on tumor prognosis based on the overall survival data from TCGA data. The TCGA RNA-seq and survival data of TCGA were downloaded from the XENA web server (https://xena.ucsc.edu/).

### Statistics.

Unless otherwise stated, each experiment was repeated at least 3 times with biologically independent samples. The numerical data are presented as the mean ± SEM as indicated in the figure legends. 2-tailed unpaired Student’s *t* tests and 1-way ANOVA with Šidák’s multiple comparisons test were used to compare the numerical data, and 2-way ANOVA with Šidák’s multiple comparisons test was used for tumor growth study with GraphPad Prism 9 (La Jolla, California, USA). Correlation was measured using Pearson’s correlation test. Survival analyses were performed using the Kaplan-Meier method with the Log-rank test for comparison. *P* values under 0.05 were considered significant.

### Study approval.

The mouse experiments were approved by IACUC of Sun Yat-sen University Cancer Center (Approval no. 2022000536). All procedures involving the collection and application of human samples were approved by the Institutional Review Boards of Sun Yat-sen University Cancer Center (Approval No. SZR2019-097), and adhered to the principles of the Declaration of Helsinki with written informed consent obtained.

### Data availability.

The raw data for RNA sequencing reported in this study is available at Genome Sequence Archive (GSA) database of National Genomics Data Center (NGDC) with the accession number CRA019697 (https://www.ngdc.cncb.ac.cn/gsa). The data authenticity of this article has also been validated by uploading the key raw data onto the Research Data Deposit platform (www.researchdata.org.cn) and approved by the Sun Yat-sen University Cancer Center Data Access/Ethics Committee with the approval number RDDB2024179513. The supporting data values for all data points in graphs are reported in the Supporting Data Values file, which is included with the online supplemental material.

## Author contributions

HZ conducted most experiments and drafted the manuscript. ZW conducted some key experiments and revised the manuscript. JW, YQZ, QZ, SH, TW, YL, LC, HG, JY, H Jin, CX, ML, JL, and XW performed parts of experiments and provided technical assistance. H Jiang, CJ, LX, and XSZ provided key reagents. XX conceived and designed the study, supervised the project, and revised the manuscript. The order of the cofirst authors has been determined on the basis of their efforts and contributions to the study.

## Supplementary Material

Supplemental data

Unedited blot and gel images

Supporting data values

## Version 1 01/16/2025

Electronic publication
