# Supplementary material for: Endothelial STING-JAK1 interaction promotes tumor vasculature normalization and antitumor immunity
Source: J Clin Invest. 2025 Jan 16;135(2):e180622. doi: 10.1172/JCI180622 (PMC11735096; doi:10.1172/JCI180622)
Supplement: Supplemental data [file jci-135-180622-s135.pdf]

Supplementary Figures and Figure Legends

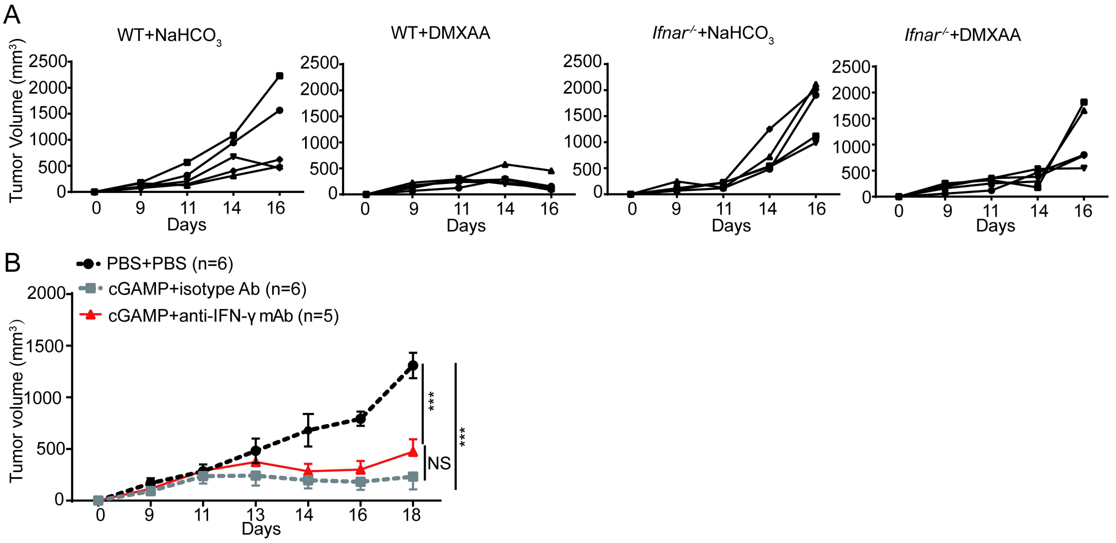

Supplementary Figure 1. STING agonist inhibits tumor growth depending on IFNAR signaling but not IFN-γ. **(A)** Tumor growth curves for each mouse after intratumoral (i.t.) injection of DMXAA (200 µg/mouse) in WT or *Ifnar*$^{-/-}$ mice bearing B16 tumors. **(B)** C57BL/6 mice bearing B16 tumors were treated when tumor size reached 100 mm^3, with i.t. injection of cGAMP (25 µg/mouse) on days 10 and 12, and intraperitoneal (i.p.) injection of anti-IFN-γ mAb (100 µg/mouse) on days 9, 12, 15. Tumor growth was monitored. The number of mice in each group was showed in the Figure. Data are represented as mean ± SEM. NS, not significant; ***P < 0.001, by 2-way ANOVA with Sidak's multiple comparisons test **(B)**.

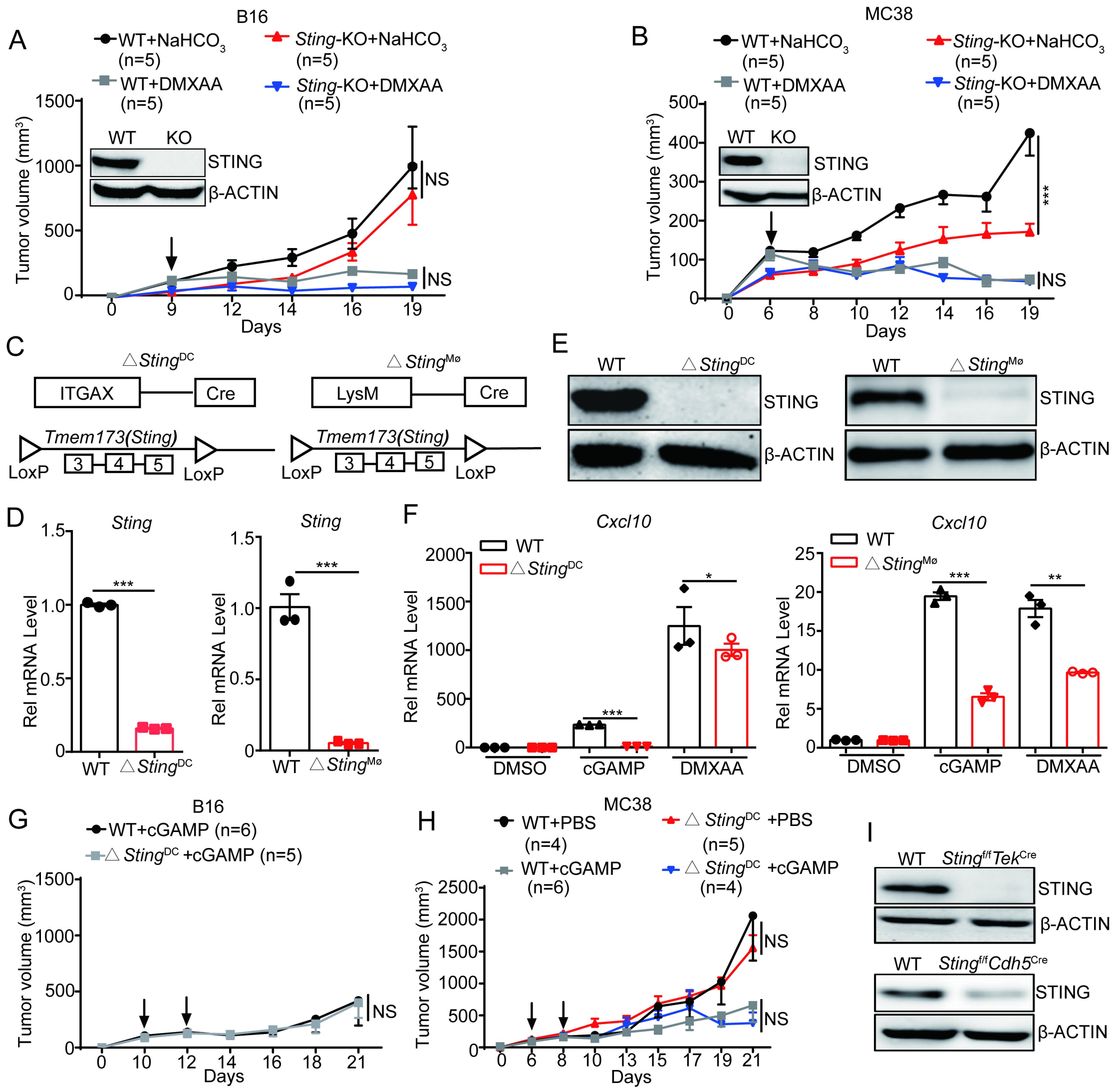

Supplementary Figure 2. STING expression in endothelial cells is essential for the antitumor effect of STING agonist on MC38 and B16 tumor models. **(A-B)** WT mice bearing WT or *Sting*-KO B16 (**A**) or MC38 (**B**) tumors were treated with i.t. injection of DMXAA (200 μg/mouse), tumor growth was recorded. $NaHCO_3$ vehicle treatment was used as control. **(C)** Schematic diagram showing *Sting*$^{f/f}$/*Itgax-Cre* (Δ*Sting*^{DC}) and *Sting*$^{f/f}$/*LysM-Cre* (Δ*Sting*$^{M\phi}$) mice with specific knockout of *Sting* in DCs and Macrophages, respectively. **(D-E)** STING deficiency was detected by qRT-PCR **(D)** and Western blot (WB, **E**) in BMDCs or BMDMs isolated from Δ*Sting*^{DC} or Δ*Sting*$^{M\phi}$ mice. **(F)** STING agonists (cGAMP and DMXAA, 5μg/ml) induced *Cxcl10* expression in BMDCs and BMDMs were detected by qRT-PCR. **(G-H）** WT or Δ*Sting*^{DC} mice bearing B16 **(G)** or MC38 **(H)** tumors were treated with i.t. injection of cGAMP (25 μg/mouse), tumor growth was recorded. PBS vehicle treatment was used as control. **(I)** STING deficiency in endothelium was detected by WB in *Sting*$^{f/f}$/*Tek-Cre* and *Sting*$^{f/f}$/*Cdh5-Cre* mice. The number of mice in each group was showed in the Figure. Data are represented as mean ± SEM. NS, not significant; *P <0.05; **P < 0.01; ***P < 0.001, by two-tailed unpaired t-test (**D**, **F**), and tumor growth curves were analyzed by 2-way ANOVA with Sidak's multiple comparisons test (**A**, **B**, **G** and **H**).

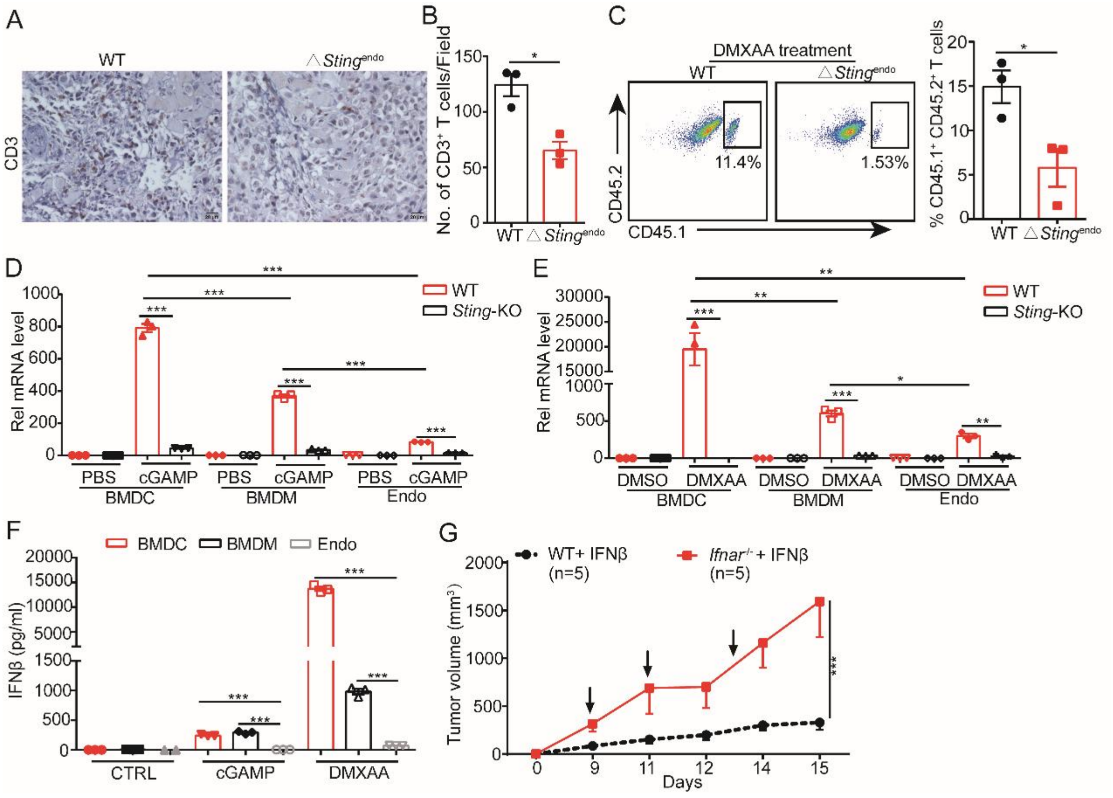

Supplementary Figure 3. Deficiency of STING in endothelial cells impairs tumor infiltration of CD8$^+$ T cells and tumor blood vessel normalization induced by STING agonist. **(A-B)** Representative images and quantified results showing intratumoral CD3$^+$ T cells infiltration detected by immunohistochemical staining after i.t. treatment of DMXAA (200 µg/mouse) on B16 tumor-bearing WT or *Sting*$^{f/f}$/*Cdh5-Cre* mice. **(C)** CD45.1$^+$ CD45.2$^+$ CD8$^+$ T cells were injected into B16 tumor-bearing CD45.2$^+$ C57BL/6 mice (WT or *Sting*$^{f/f}$/*Tek-Cre*) via the tail vein and DMXAA (200 µg/mouse) was injected by intratumoral injection; 3 days later, CD45.1$^+$ CD45.2$^+$ CD8$^+$ T cell infiltrations in the tumors were detected by FACS. **(D-E)** qPCR detection of *Ifnβ* expression in DCs, macrophages and endothelial cells after treatment with cGAMP (5 µg/ml, **D**) or DMXAA (5 µg/ml, **E**) for 3 hours. **(F)** ELISA detection of supernatant IFNβ levels in BMDCs, BMDMs and endothelial cells after treatment with STING agonists (cGAMP and DMXAA, 5 µg/ml) for 24 hours. **(G)** B16 tumor-bearing WT or *Ifnar*-KO mice received i.t. injection of IFNβ (50 ng/mouse) at the indicated time (arrows), then the tumor size was recorded. Data are represented as mean ± SEM. *P <0.05; **P < 0.01; ***P < 0.001, by two-tailed unpaired t-test **(B, C)**, or by one-way ANOVA with Sidak's multiple comparisons test **(D-F)** and tumor growth curves were used 2-way ANOVA with Sidak's multiple comparisons test **(G)**.

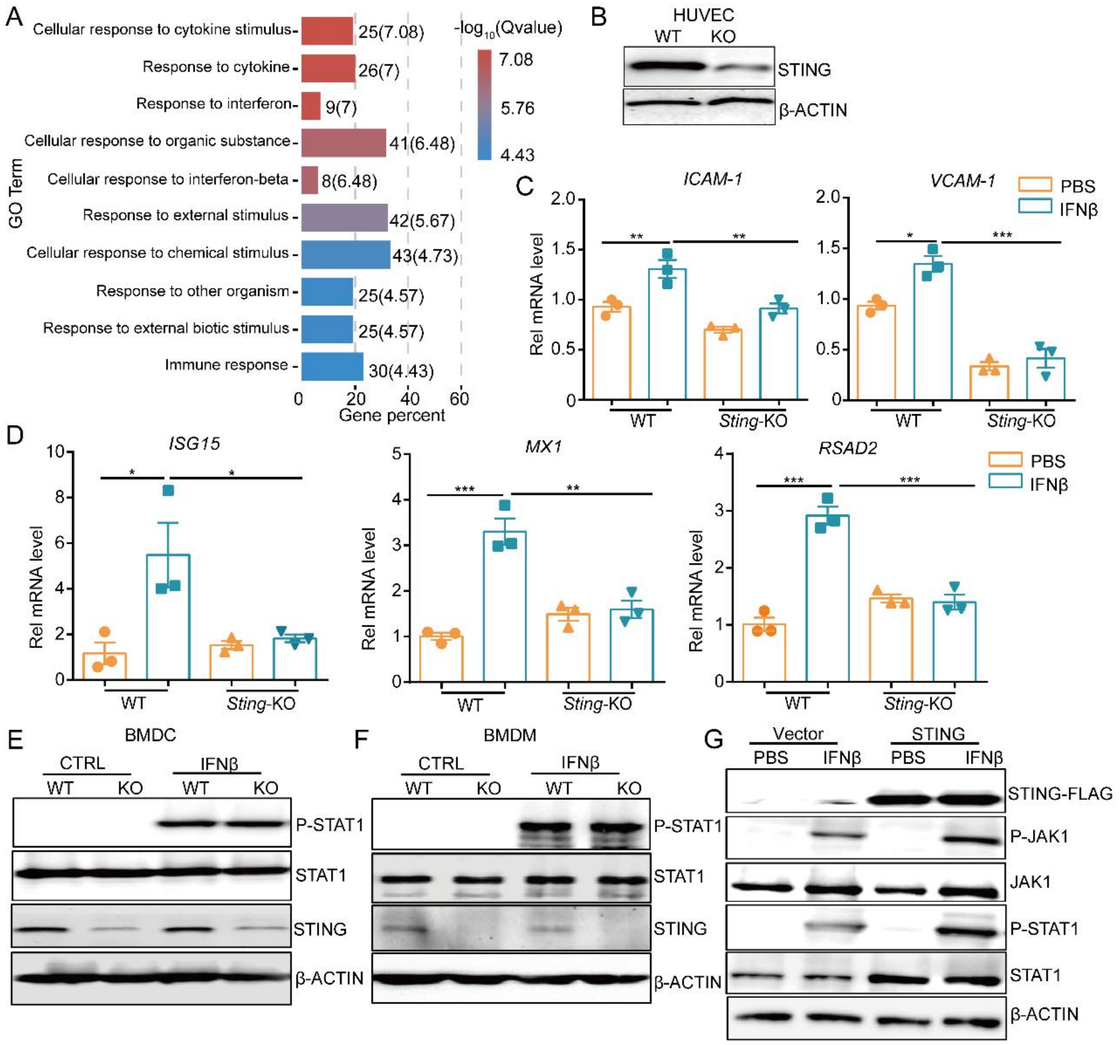

Supplementary Figure 4. STING expression in endothelial cells is required for IFNAR downstream signaling activation. **(A)** Gene ontology (GO) enrichment analysis showed significant enrichment for the response to interferon and immune response after IFNβ (10 ng/ml) stimulation for 3 hours. **(B)** STING protein expression levels were detected by WB in HUVEC cells. **(C-D)** qRT-PCR detection of the mRNA expression levels of *ICAM-1*, *VCAM-1*, *ISG15*, *MX1* and *RSAD2* in WT and *Sting*-KO HUVEC cells after IFNβ (10 ng/ ml) stimulation for 3 hours. **(E-F)** WB analysis of STAT1 phosphorylation levels in BMDCs (WT, *Sting*-KO) and BMDMs (WT, *Sting*-KO) after treatment with PBS or IFNβ (10 ng/ml) for 15 minutes. **(G)** Primary mouse endothelial cells were infected with lentivirus expressing WT STING, then treated with IFNβ (10 ng/ml) for 15 minutes, then WCL were harvested for WB analysis of phosphorylated and total JAK1, STAT1 levels (P-JAK1, JAK1, P-STAT1, STAT1). Data are represented as mean ± SEM. *P <0.05; **P < 0.01; ***P < 0.001, by one-way ANOVA with Sidak's multiple comparisons test **(C-D)**.

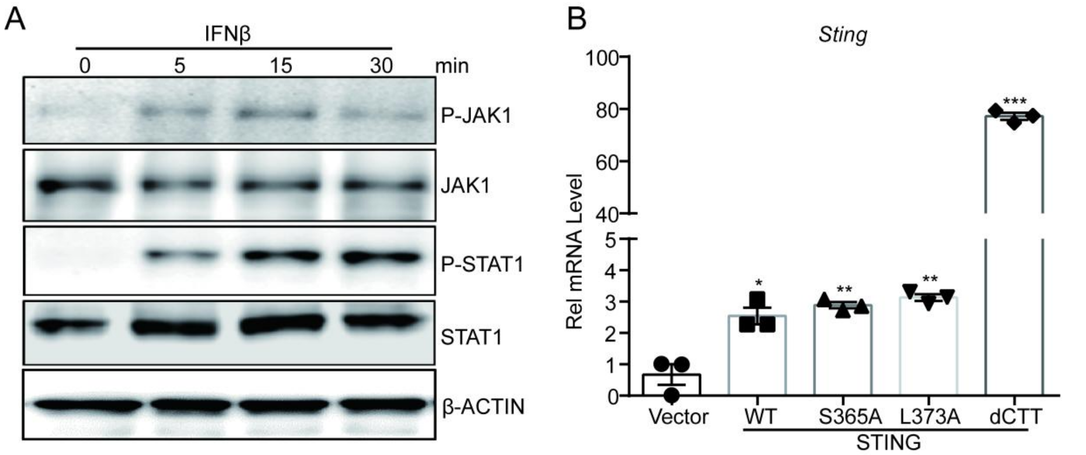

Supplementary Figure 5. STING interacts with JAK1 in primary mouse endothelial cells upon IFNβ stimulation. **(A)** Phosphorylation levels of JAK1 and STAT1 in primary mouse endothelial cells after IFNβ (10 ng/ml) stimulation at the indicated time (0, 5, 15, 30 minutes) were detected by WB. **(B)** qPCR analysis of the mRNA levels of STING in expression primary mouse endothelial cells after overexpression of WT or mutant STING. WT: WT-STING, S365A: serine 365-to-alanine, L373A: leucine 373-to-alanine, dCTT: deficiency of CTT domain. Data are represented as mean ± SEM. *P <0.05; **P < 0.01; ***P < 0.001, by two-tailed unpaired t-test **(B)**.

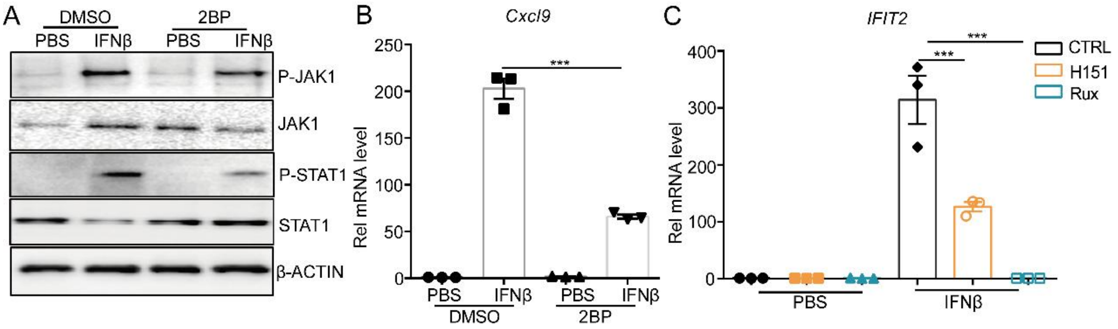

Supplementary Figure 6. STING C91A mutation disrupts its interaction with JAK1 and impairs JAK1 activation upon IFNβ stimulation. **(A)** WB analysis of phosphorylation level of JAK1 and STAT1 in mouse endothelial cells stimulated with IFNβ (10 ng/ml) for 15 minutes, with or without pre-treatment with 2-Bromopalmitate (2-BP, 10 μM). **(B)** qPCR analysis of the expression of *Cxcl9* in mouse endothelial cells stimulated with IFNβ (10 ng/ml) for 3 hours, with or without pre-treatment with 2-BP (10 μM). **(C)** qPCR analysis of the expression of *IFIT2* in HUVEC cells stimulated with IFNβ (10 ng/ml) for 3 hours, with pre-treatment of H151 (10 μg/ml) or Ruxolitinib (Rux, 5 μM) for 30 minutes. Data are represented as mean ± SEM. ***P < 0.001, by one-way ANOVA with Sidak's multiple comparisons test **(B-C)**.

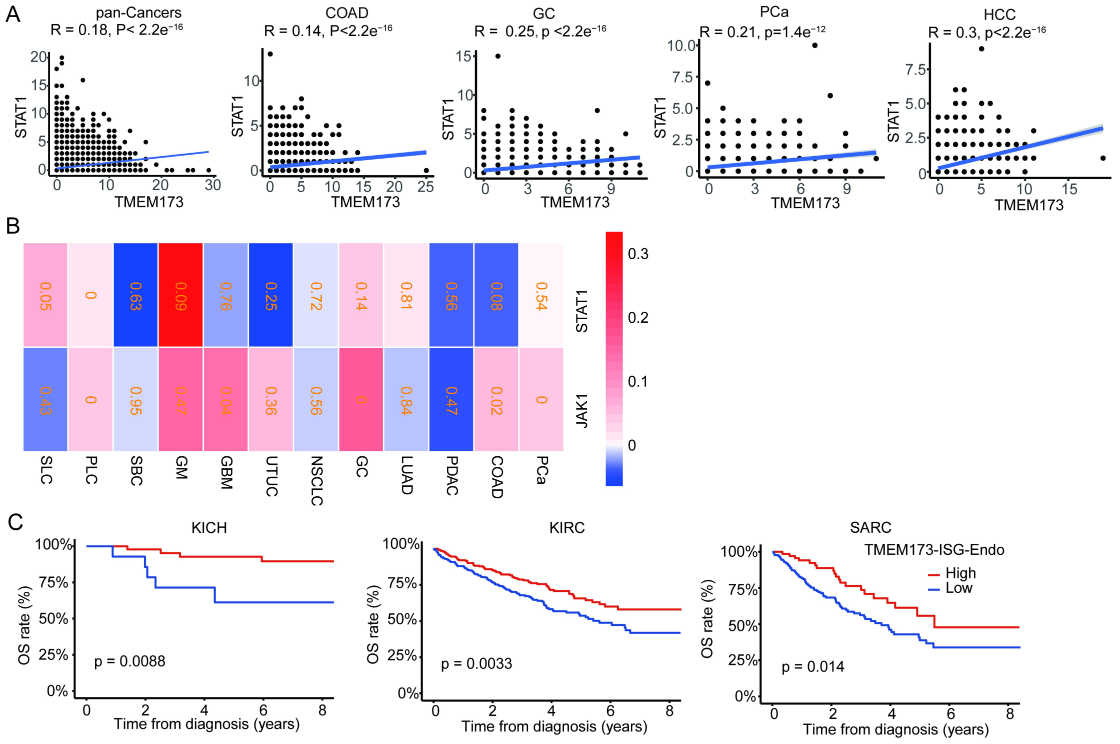

Supplementary Figure 7. Endothelial STING expression positively correlates with endothelial JAK1-STAT1 signaling and antitumor immunity. **(A)** Correlation between endothelial STING expression and endothelial STAT1 expression in pan-Cancers and COAD, GC, PCa and HCC cancers. **(B)** Correlation between endothelial STING expression and endothelial JAK1, STAT1 expression analyzed from single-cell database. **(C)** Kaplan-Meier survival curves of KICH, KIRC and SARC patients stratified by endothelial STING-ISG expression levels. COAD: colon adenocarcinoma; GC: gastric carcinoma; PCa: prostate cancer; HCC: hepatocellular carcinoma; SLC: secondary liver cancer; PLC:primary liver cancer; SBC: skull base chordoma; GM: glioblastoma; GBM: glioblastoma multiforme; UTUC: upper tract urothelial carcinoma; NSCLC: non-small cell lung cancer; LUAD: lung adenocarcinoma; PDAC: pancreatic ductal adenocarcinoma; KICH: kidney chromophobe; KIRC:kidney renal clear cell carcinoma; SARC: soft tissue sarcoma. R and P values by Pearson's correlation test **(A-B)**, P values by the log-rank test **(C)**.
