# Supplementary material for: Endothelial STING-JAK1 interaction promotes tumor vasculature normalization and antitumor immunity
Source: J Clin Invest. 2025 Jan 16;135(2):e180622. doi: 10.1172/JCI180622 (PMC11735096; doi:10.1172/JCI180622)

Figure 4G

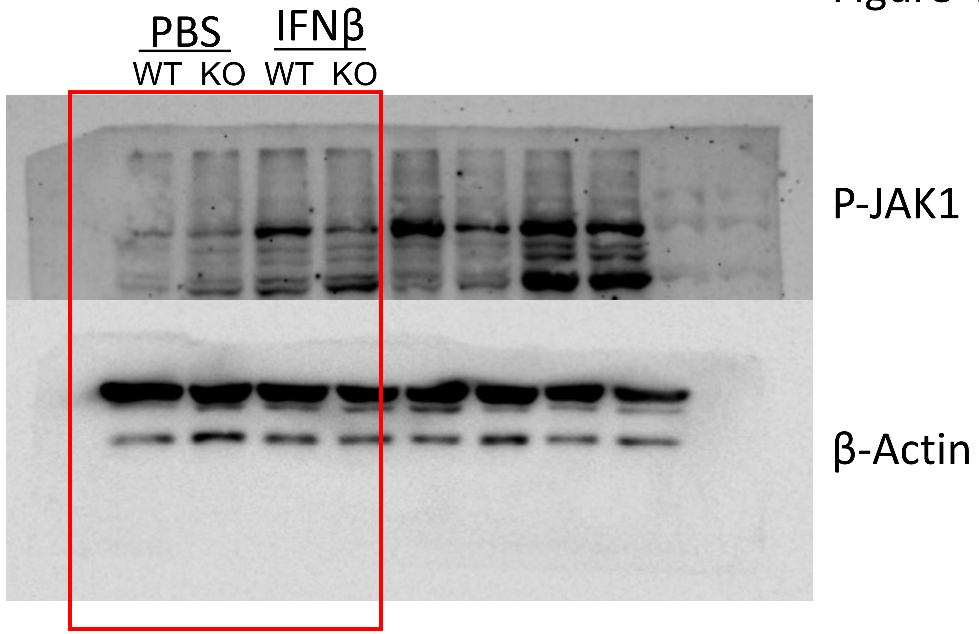
PBS
IFNβ
WT KO WT KO
P-JAK1
β-Actin

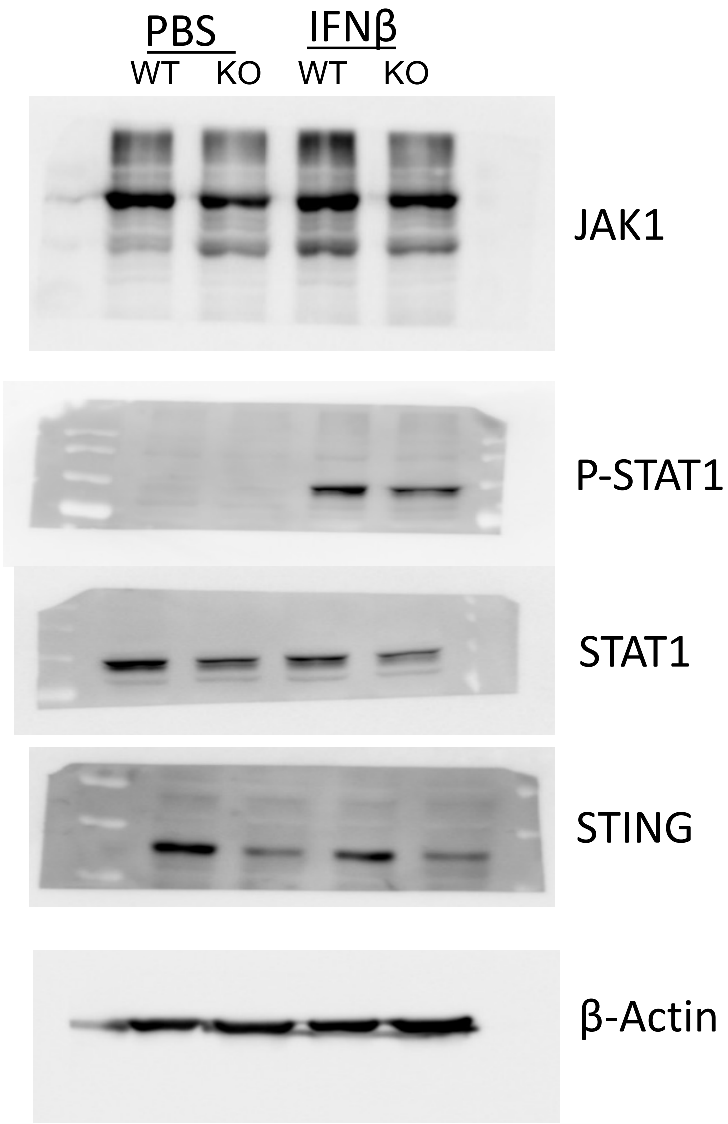
PBS
IFNβ
WT KO WT KO
JAK1
P-STAT1
STAT1
STING
β-Actin

Figure 5A

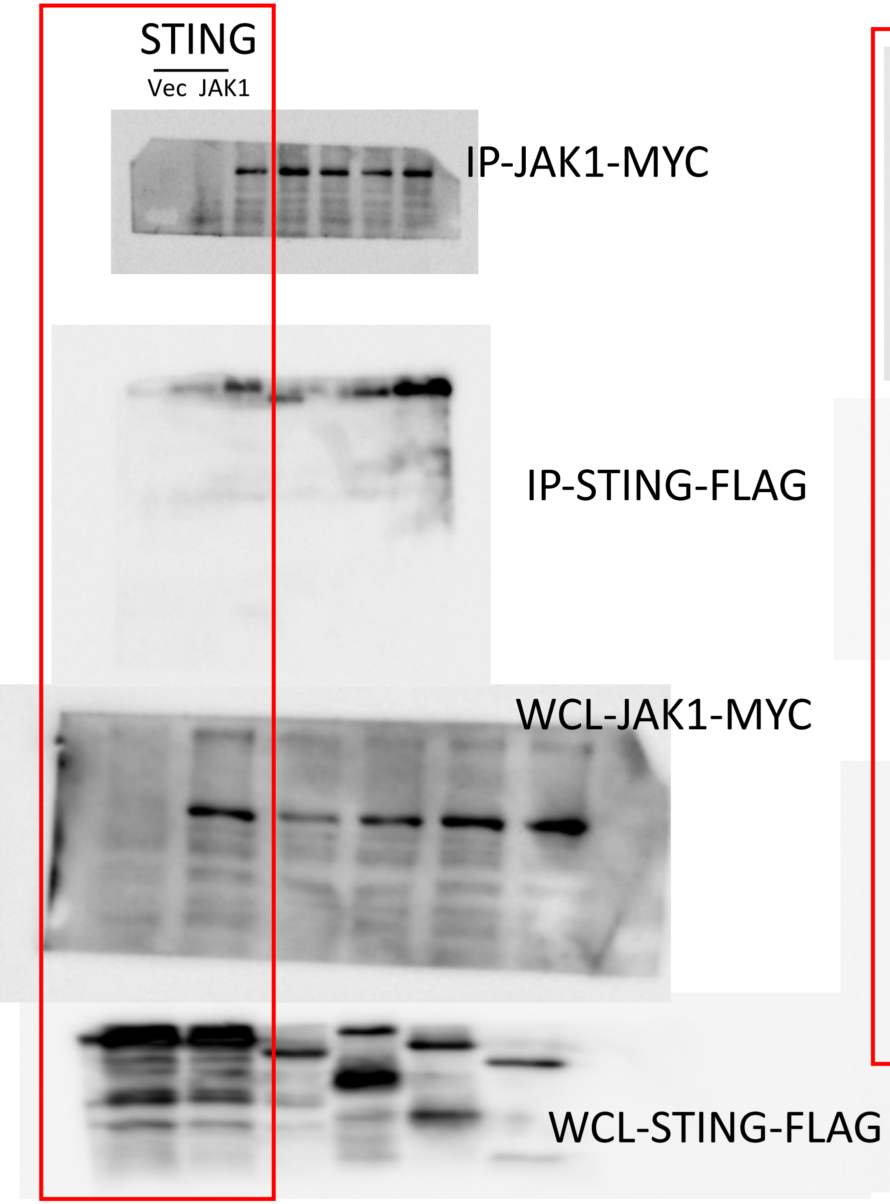

Figure 5B

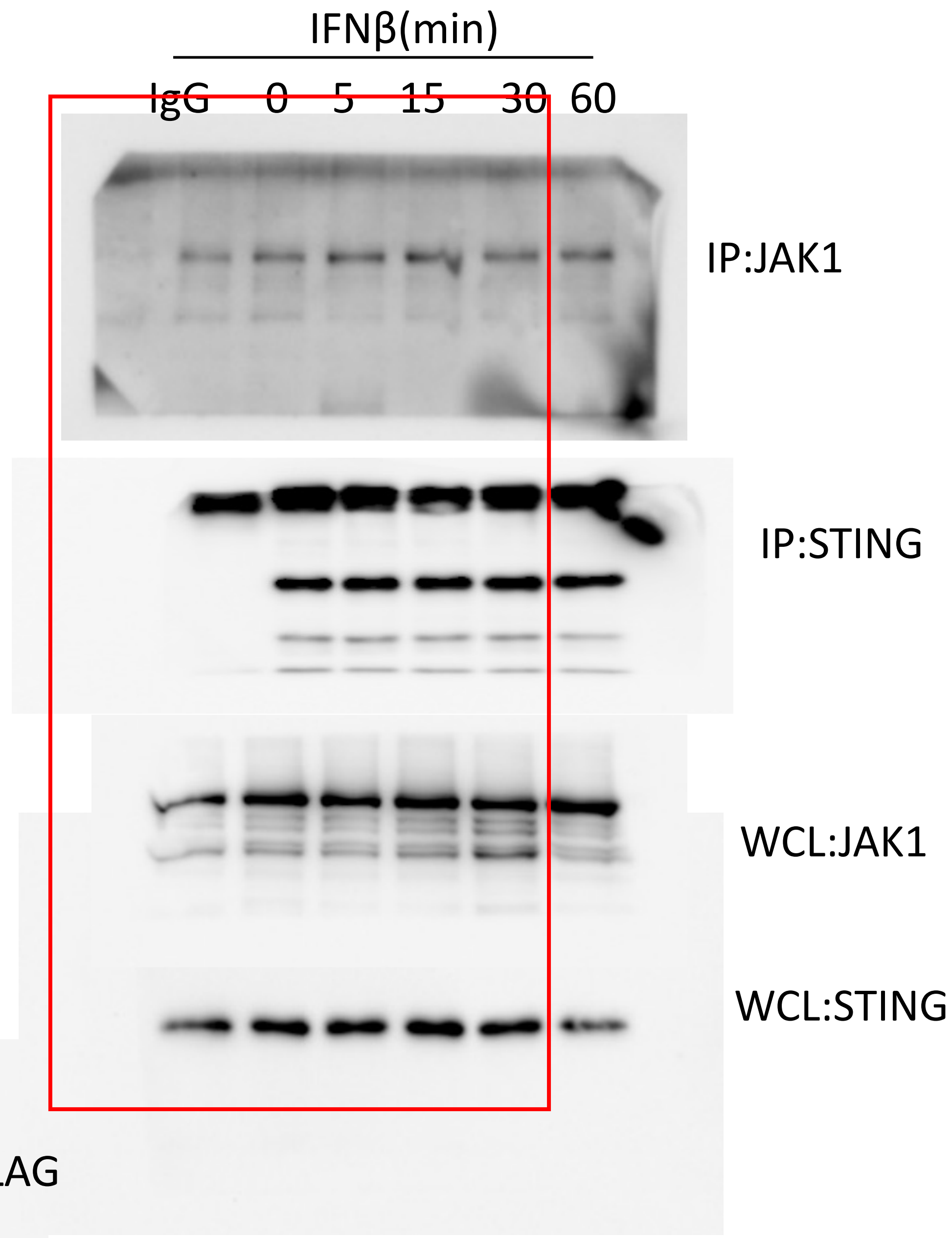

Figure 5C

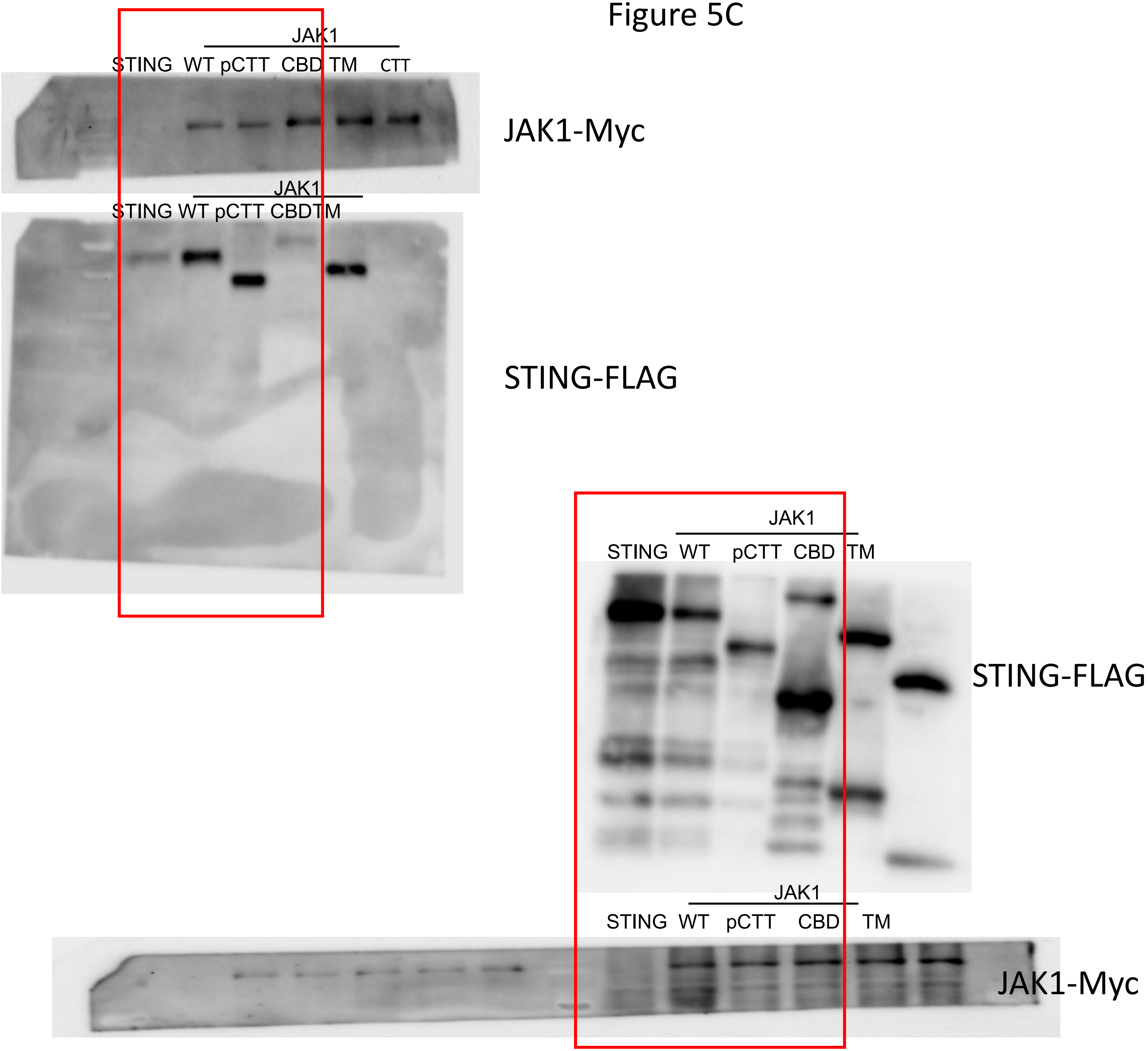
JAK1
STING WT pCTT CBD TM CTT
JAK1-Myc
JAK1
STING WT pCTT CBDTM
STING-FLAG
JAK1
STING WT pCTT CBD TM
STING-FLAG
JAK1
STING WT pCTT CBD TM
JAK1-Myc

Figure 5D

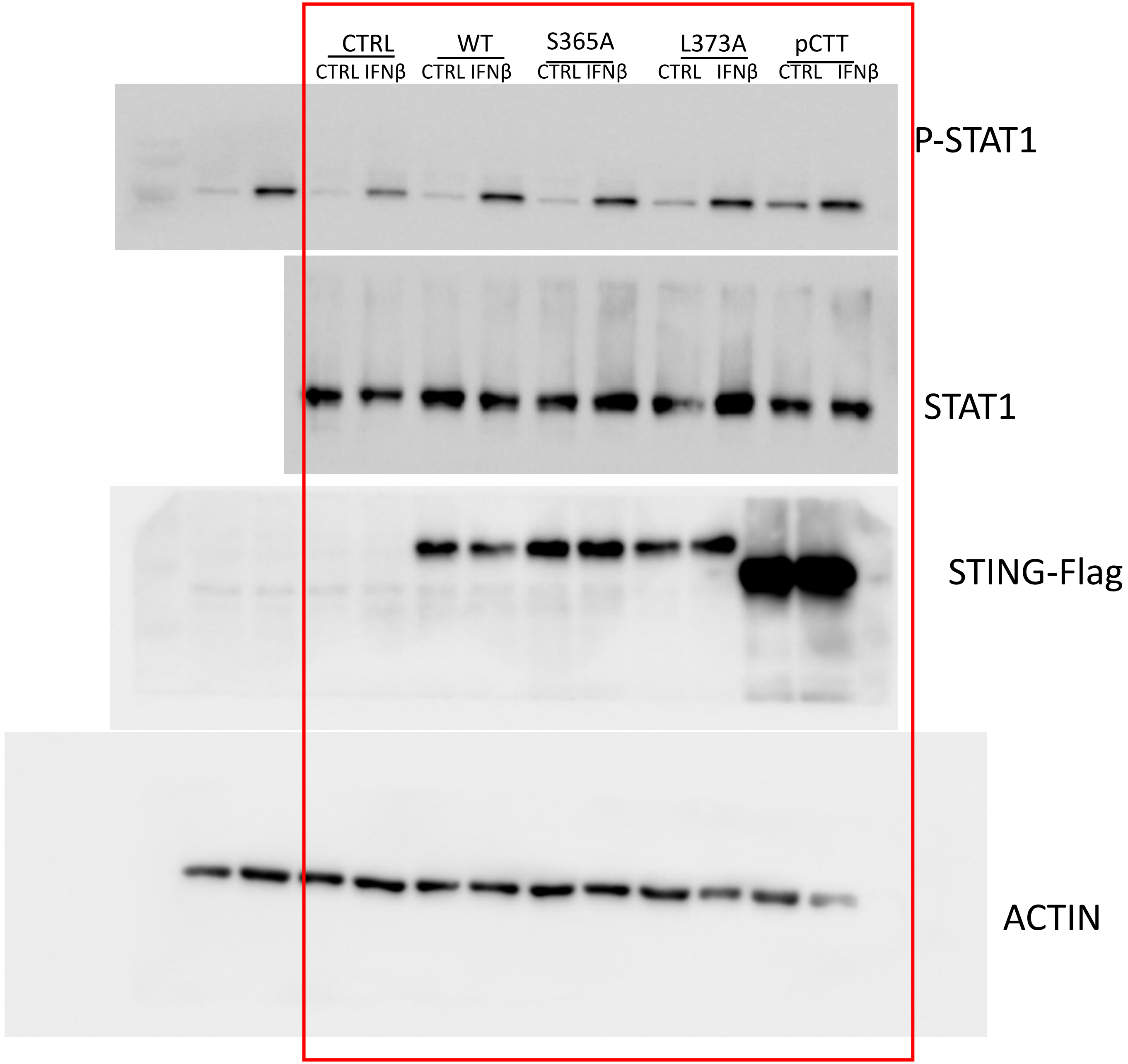

CTRL
WT
S365A
L373A
pCTT
CTRL IFNβ
CTRL IFNβ
CTRL IFNβ
CTRL IFNβ
CTRL IFNβ
P-STAT1
STAT1
STING-Flag
ACTIN

Figure 5F

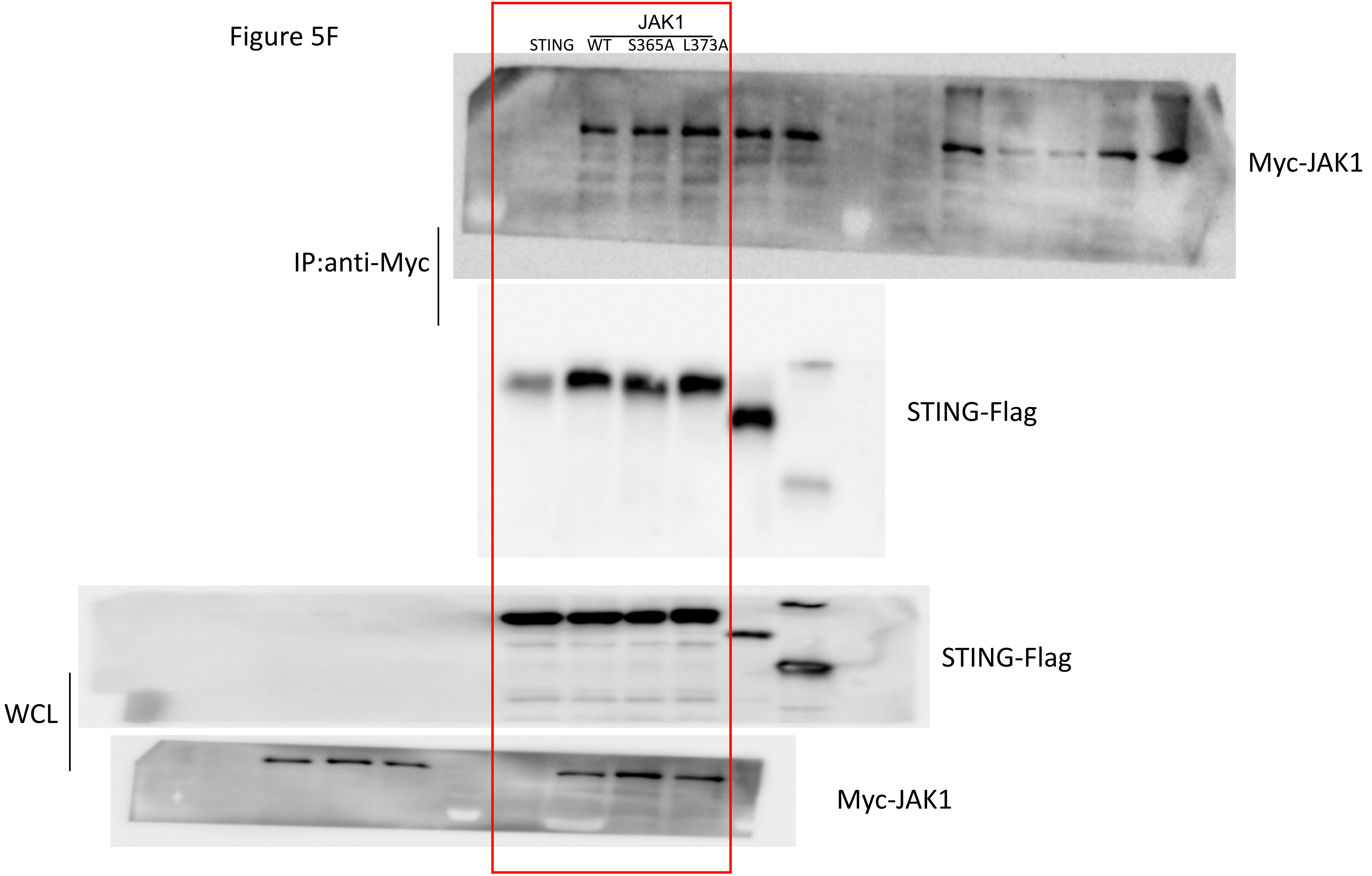
JAK1
STING
WT
S365A
L373A
Myc-JAK1
IP:anti-Myc
STING-Flag
STING-Flag
WCL
Myc-JAK1

Figure 6A

Figure 6B

Primary endothelial cells

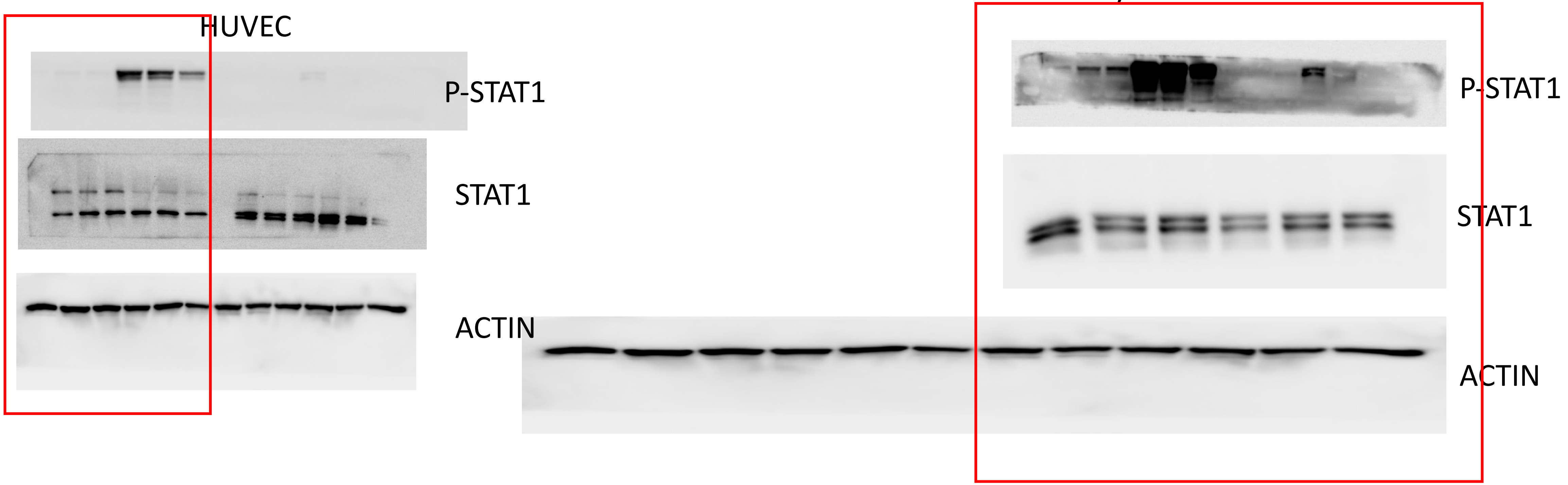

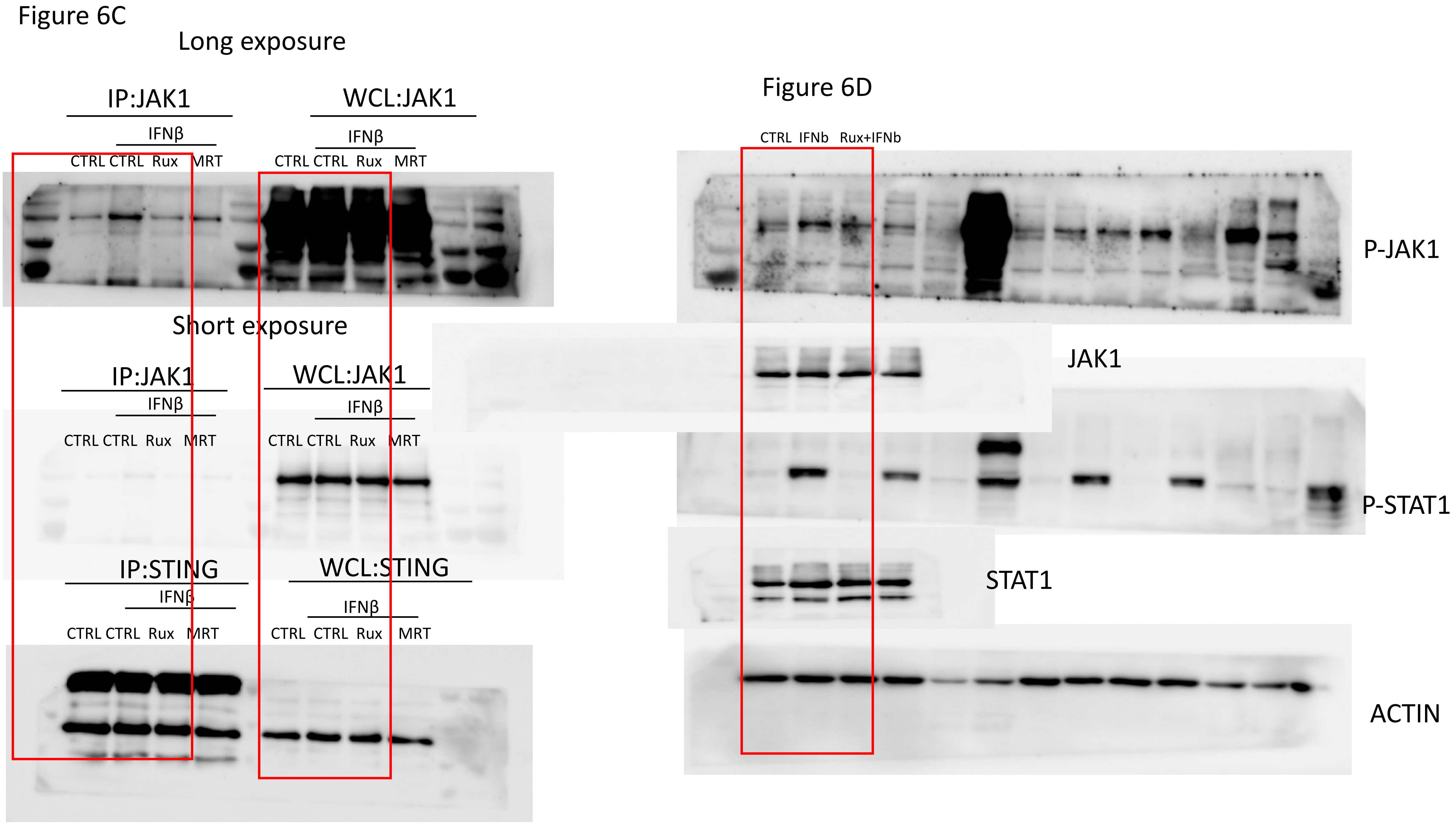
Figure 6C
Long exposure
IP:JAK1
IFNβ
CTRL CTRL Rux MRT
WCL:JAK1
IFNβ
CTRL CTRL Rux MRT
Short exposure
IP:JAK1
IFNβ
CTRL CTRL Rux MRT
WCL:JAK1
IFNβ
CTRL CTRL Rux MRT
IP:STING
IFNβ
CTRL CTRL Rux MRT
WCL:STING
IFNβ
CTRL CTRL Rux MRT
Figure 6D
CTRL IFNb Rux+IFNb
P-JAK1
JAK1
P-STAT1
STAT1
ACTIN

Figure 6E

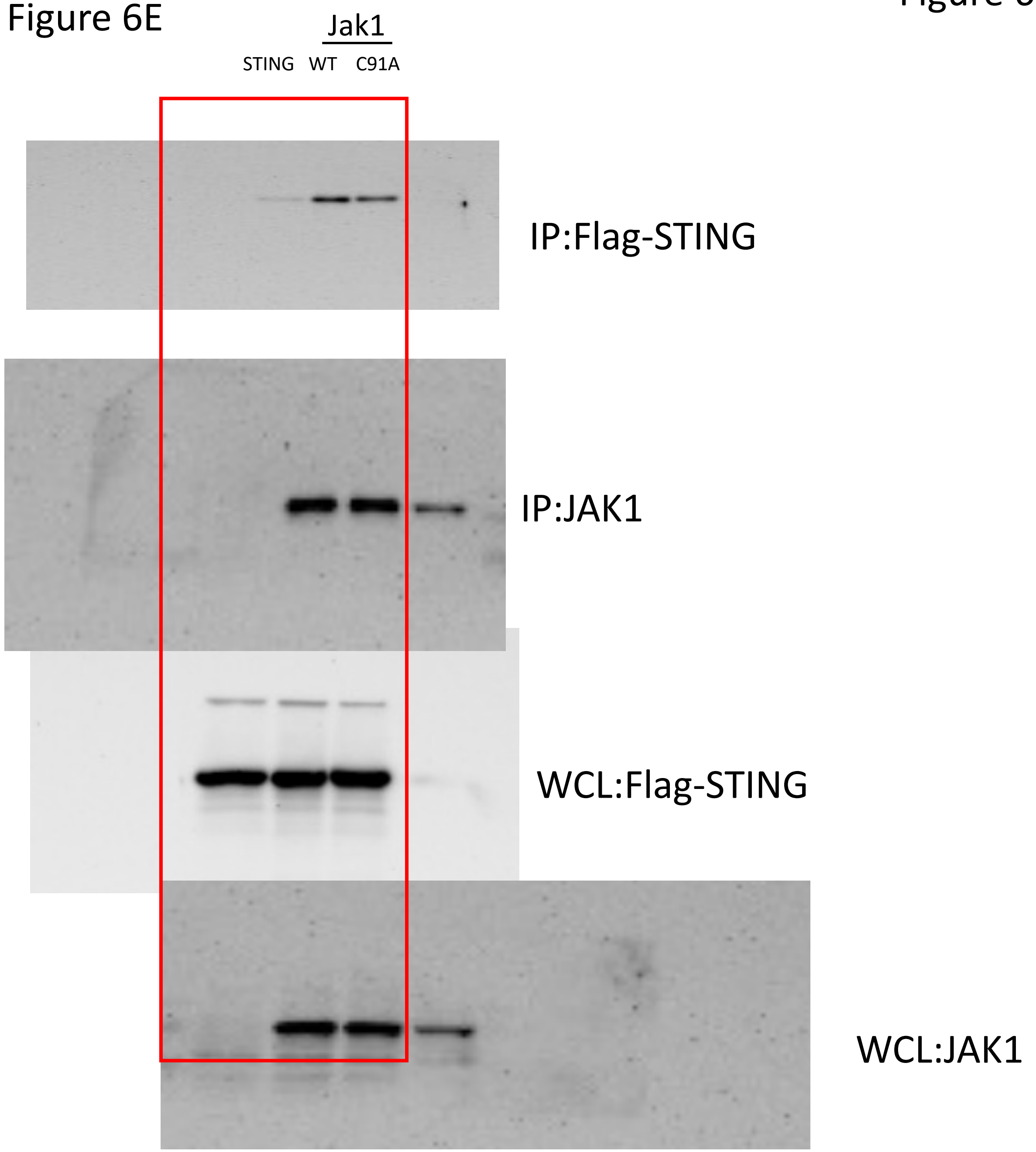

Figure 6H

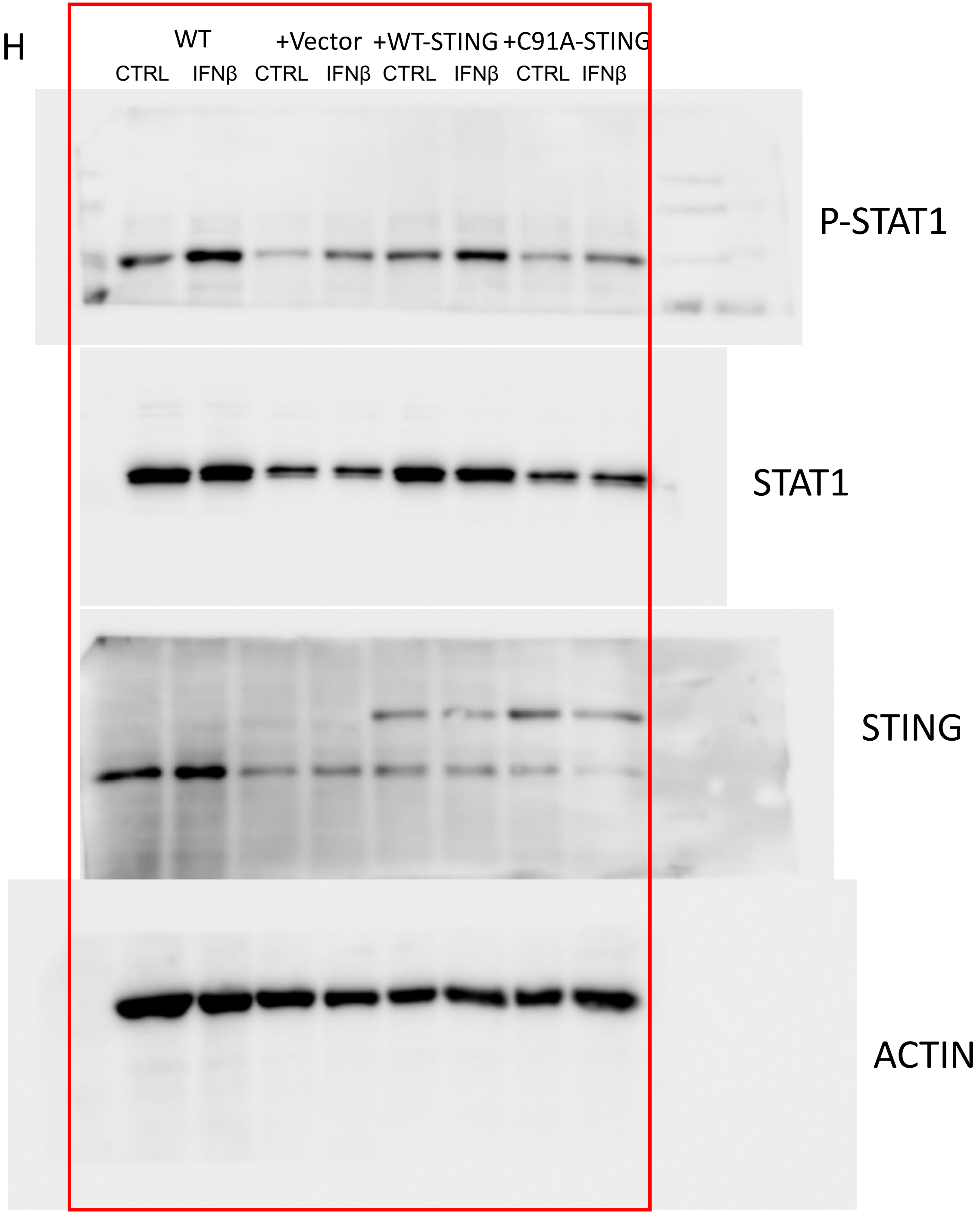

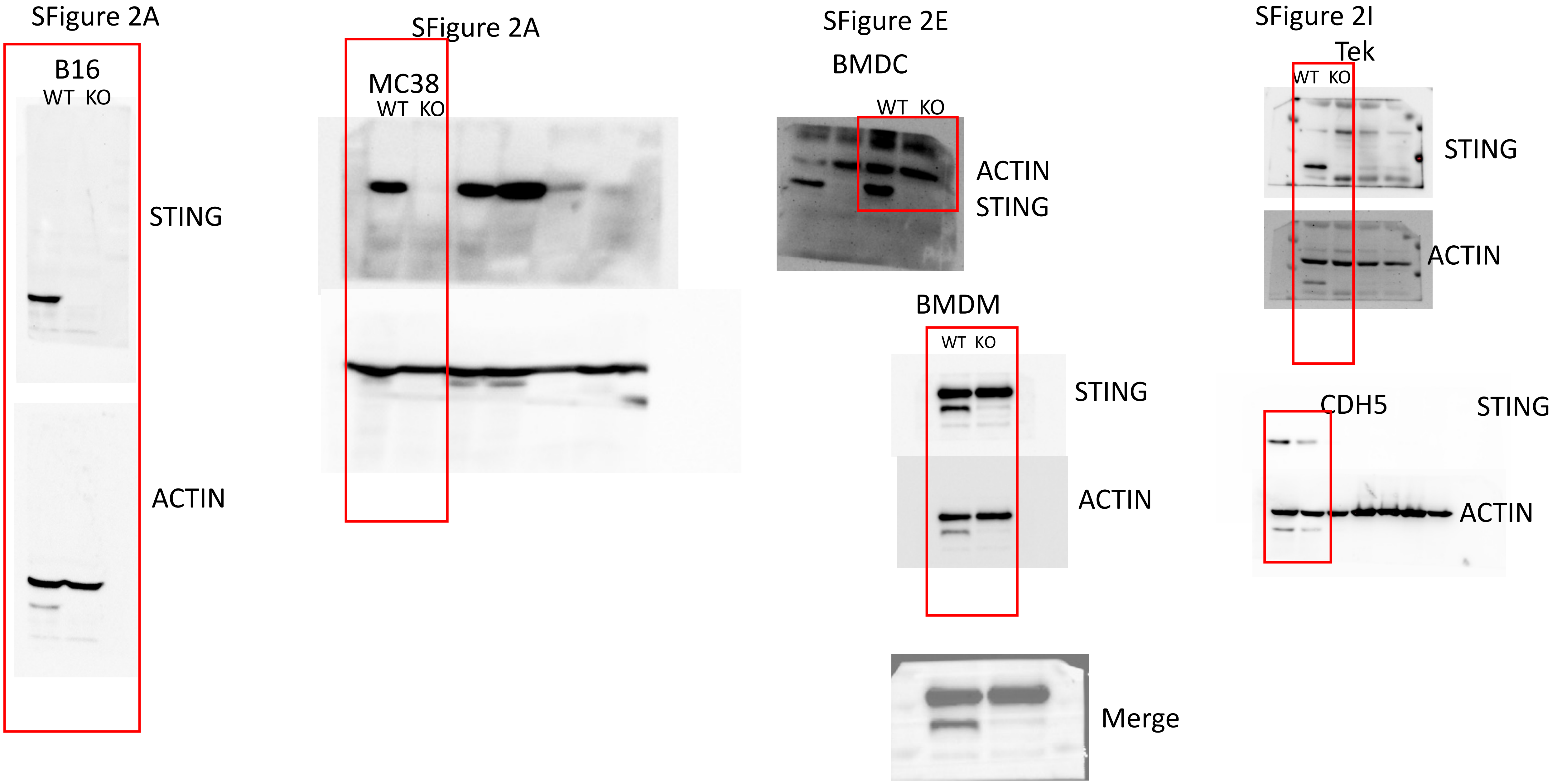
SFigure 2A
B16
WT KO
STING
ACTIN
SFigure 2A
MC38
WT KO
SFigure 2E
BMDC
WT KO
ACTIN
STING
BMDM
WT KO
STING
ACTIN
Merge
SFigure 2I
Tek
WT KO
STING
ACTIN
CDH5
STING
ACTIN

Sfigure 4B

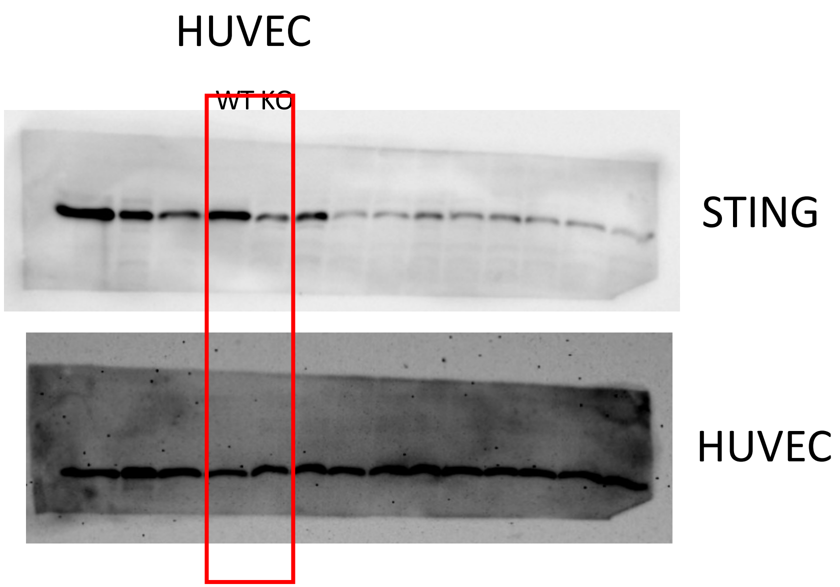

Sfigure 4E 20210111

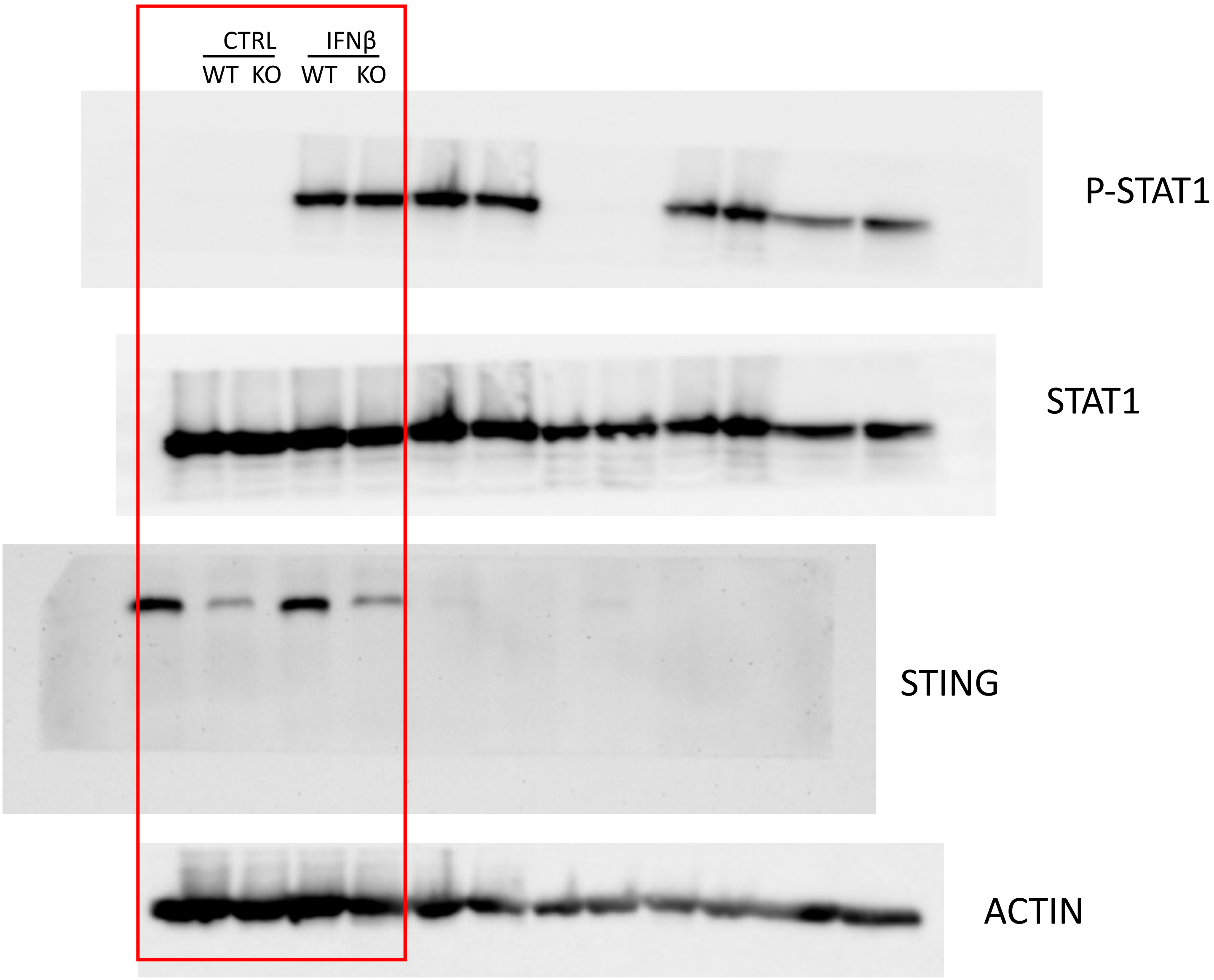

Sfigure 4F

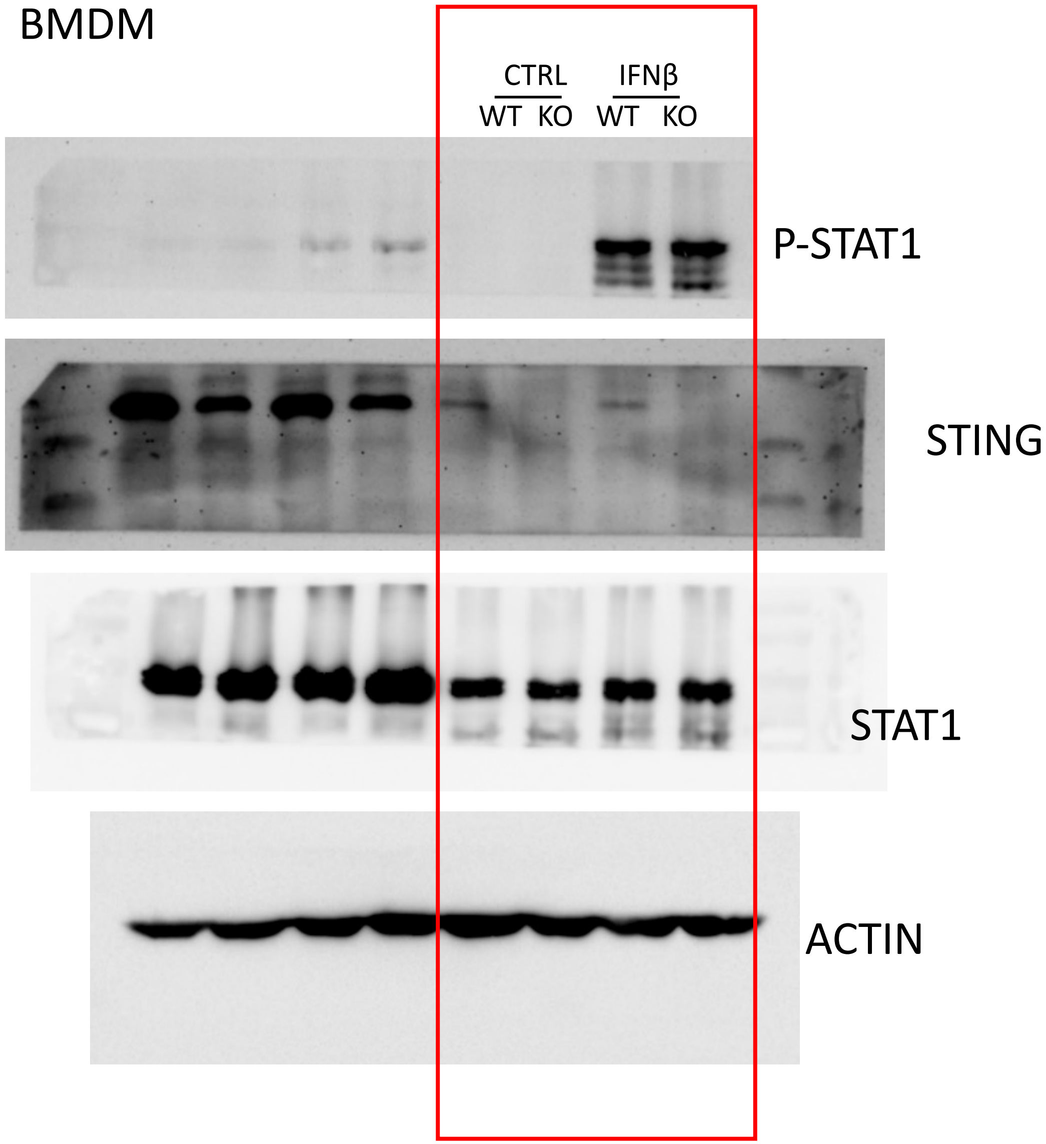

Sfigure 4G

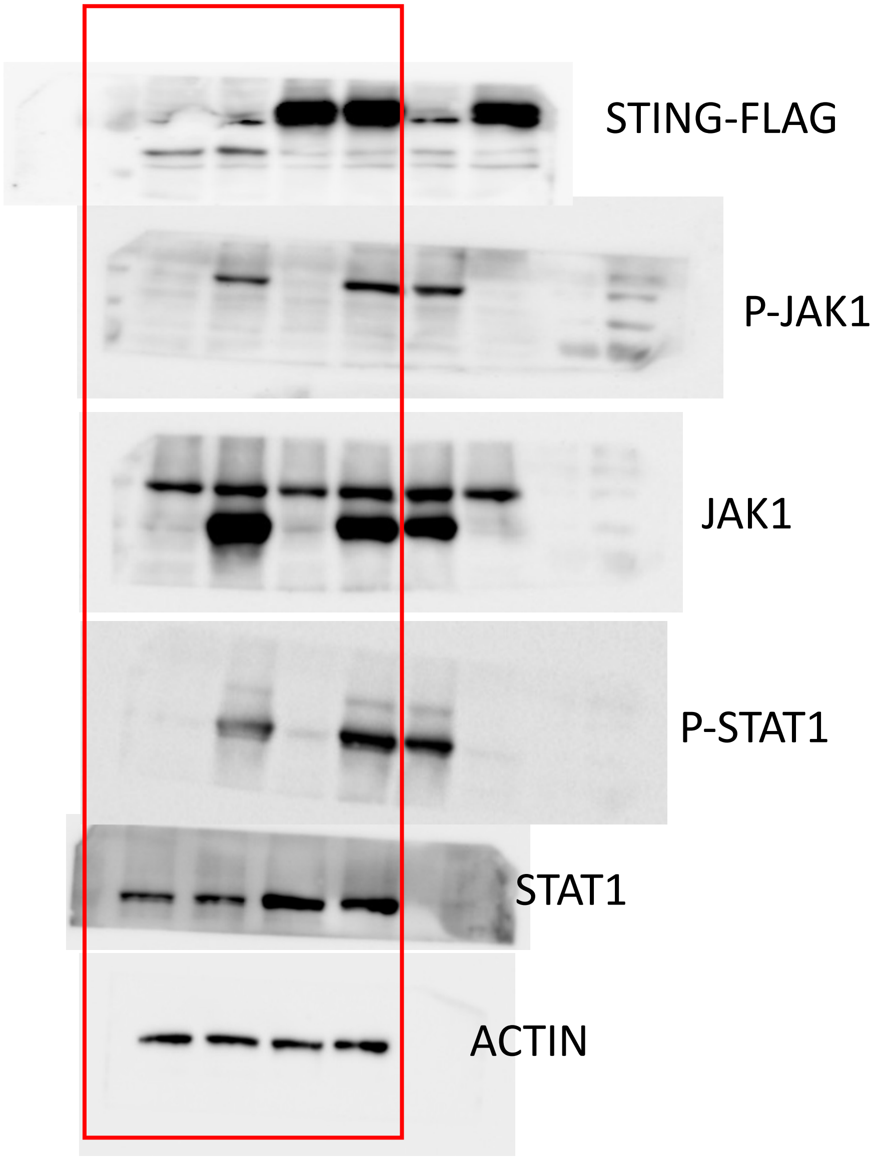

Sfigure 5A 2023.02.01

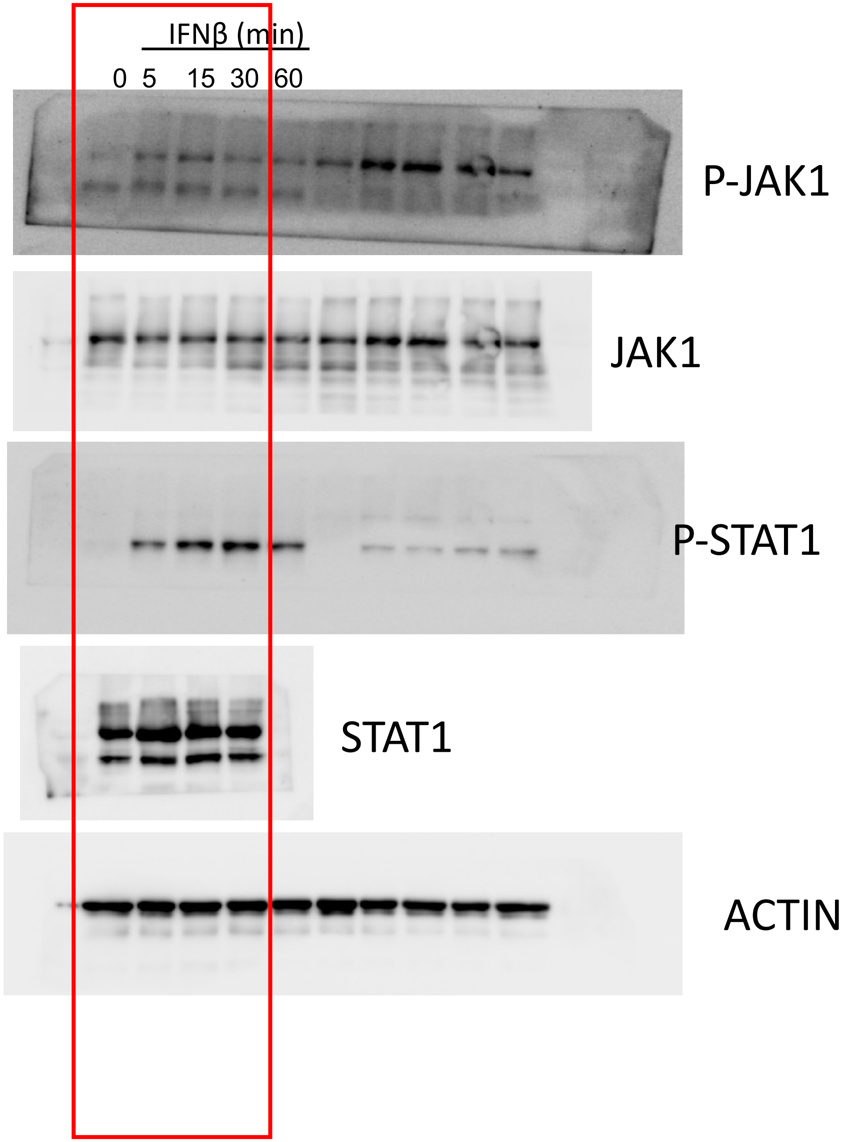

Sfigure 6A

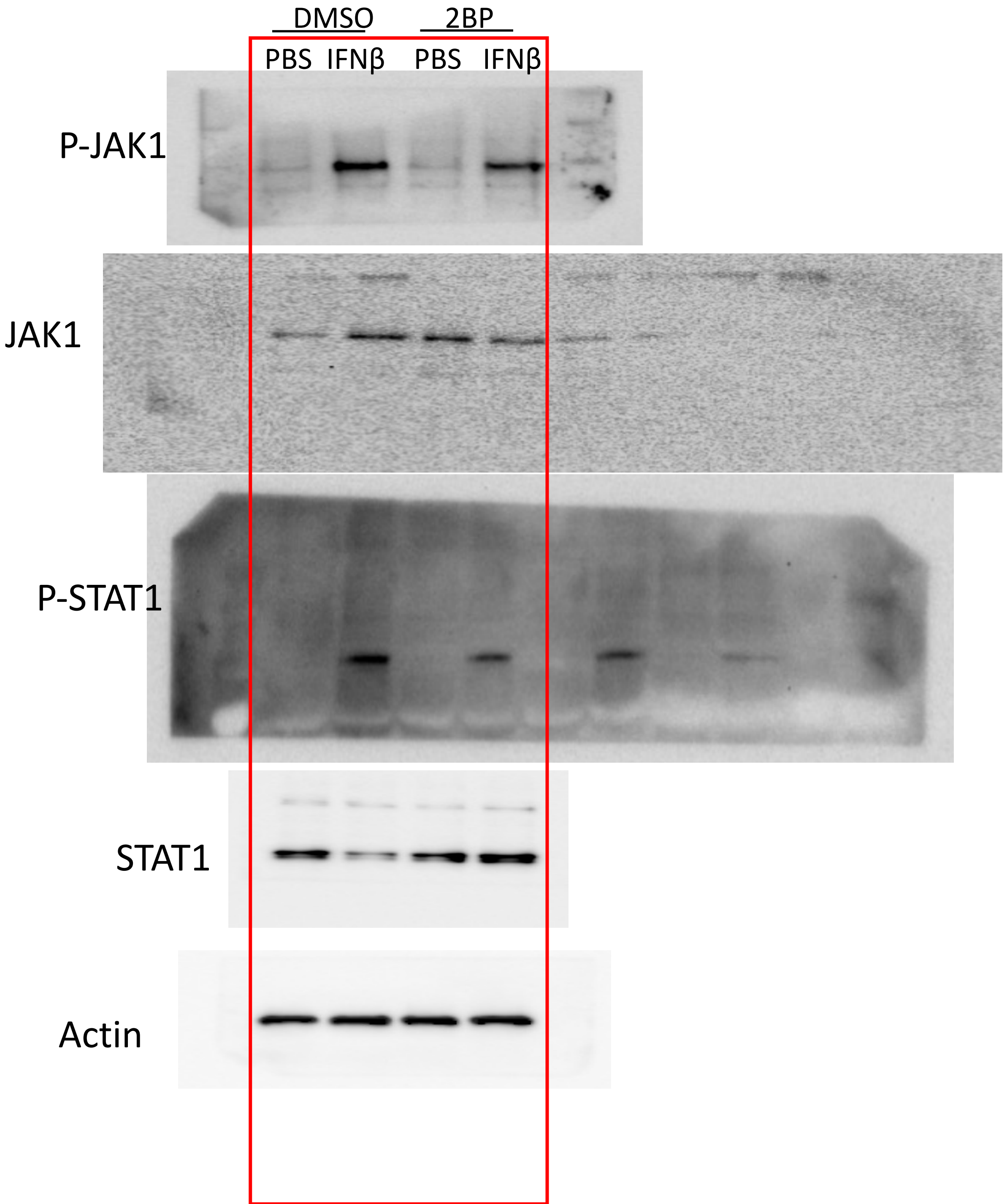

Supplement: Unedited blot and gel images [file jci-135-180622-s136.pdf]
